# Childhood Maltreatment and Cognitive Functioning in Bipolar Disorder: A Systematic Review and Meta‐Analysis

**DOI:** 10.1111/acps.13813

**Published:** 2025-05-06

**Authors:** Natalia E. Fares‐Otero, Anaid Pérez‐Ramos, Ricardo Lopez‐Escribano, Sara Martin‐Parra, Luis Alameda, Sarah L. Halligan, Kamilla Woznica Miskowiak, Eduard Vieta

**Affiliations:** ^1^ Bipolar and Depressive Disorders Unit, Department of Psychiatry and Psychology Hospital Clínic, Institute of Neurosciences (UBNeuro), University of Barcelona (UB) Barcelona Spain; ^2^ Fundació Clínic per a la Recerca Biomèdica (FCRB), Institut d'Investigacions Biomèdiques August Pi i Sunyer (IDIBAPS) Barcelona Spain; ^3^ Network Centre for Biomedical Research in Mental Health (CIBERSAM), Health Institute Carlos III (ISCIII) Barcelona Spain; ^4^ Neuropsychopharmacology and Psychobiology Research Group, Department of Neuroscience Faculty of Medicine, University of Cádiz Cádiz Spain; ^5^ Department of Psychiatry University Hospital Principe de Asturias Alcala de Henares Spain; ^6^ Department of Psychosis Studies Institute of Psychiatry, Psychology and Neuroscience, King's College London London UK; ^7^ National Psychosis Unit South London and Maudsley NHS Foundation Trust London UK; ^8^ Service of General Psychiatry, Treatment and Early Intervention in Psychosis Program Lausanne University Hospital (CHUV) Lausanne Switzerland; ^9^ Department of Psychology University of Bath Bath UK; ^10^ Department of Psychiatry Faculty of Medicine and Health Sciences, Stellenbosch University Stellenbosch South Africa; ^11^ Department of Psychiatry and Mental Health University of Cape Town Cape Town South Africa; ^12^ Neurocognition and Emotion in Affective Disorders (NEAD) Centre, Psychiatric Centre Copenhagen, Frederiksberg Hospital Copenhagen Denmark; ^13^ Department of Psychology University of Copenhagen Copenhagen Denmark; ^14^ Department of Medicine School of Medicine & Health Sciences, University of Barcelona (UB) Barcelona Spain

**Keywords:** attention, childhood trauma, executive functions, intelligence, memory, mood disorder, neglect, neurocognition, social cognition, traumatic stress

## Abstract

**Aims:**

Characterising the association between childhood maltreatment (CM) and cognitive functioning in bipolar disorder (BD) is crucial for improving the understanding of how early environmental risk factors impact the presentation of the disorder. We conducted a systematic review and meta‐analysis to estimate associations between overall and subtypes of CM, global cognition/IQ, and five cognitive domains in BD (attention/processing speed, verbal memory/learning, working memory, executive functions/verbal fluency, and social cognition), and to explore moderators/mediators in these associations.

**Methods:**

A systematic search was performed on 24 June 2024 to identify published peer‐reviewed articles in six databases (PROSPERO‐CRD42023468641).

**Results:**

From 780 identified records, 20 studies were included, comprising 2457 individuals with BD (M ± SD, age in years = 39.5 ± 9.7; 41.3% males; BD type I = 81.2%); 152 effect sizes were pooled in random‐effect meta‐analyses. Overall CM was negatively associated with global cognition/IQ, attention/processing speed, and verbal memory/learning (*r* = −0.14 to −0.18, *p* = 0.002 to < 0.001). Sexual/physical abuse and physical neglect were negatively associated with global cognition/IQ, working memory, and executive functions/verbal fluency (*r* = −0.07 to −0.18, *p* = 0.037 to < 0.001). Emotional abuse was negatively associated with working memory (*r* = −0.12, *p* = 0.002). Emotional neglect was unrelated to cognitive functions. CM (overall/subtypes) was unrelated to social cognition. Meta‐regressions did not identify any consistent moderators. Narrative synthesis identified possible moderators/mediators. Associations were of small magnitude, and a limited number of studies assessing CM subtypes and cognitive functions are available.

**Conclusion:**

CM exposure is associated with worse cognitive performance in people with BD, an effect observed across multiple maltreatment types and cognitive domains. Besides trauma‐informed interventions, those with BD and CM require cognitive assessment and therapies to rehabilitate cognitive functioning.

**Summary**

- Summations
  ○ Being exposed to CM, especially physical maltreatment, is associated with worse cognitive functioning in BD.
  ○ There is no evidence of an association between CM and social cognition of emotion perception and theory of mind skills in BD.
  ○ There is little evidence of moderating/mediating factors: cognitive disruptions increase with higher depression symptom severity, and lower IQ may be an underlying mechanism between CM and cognitive problems in BD.
- Limitations
  ○ The number of relevant studies is small, with different cognitive tasks across studies. Consequently, analyses of heterogeneity and moderators are limited.
  ○ Evidence of moderators and mediators of the pathway between CM and cognitive outcomes in BD is scarce.
  ○ Evidence relating to CM (overall and subtypes) and social cognition is particularly limited, and investigation of associations with cognitive features in BD is urgently needed.

## 1 Introduction

Bipolar disorder (BD) is a leading cause of years lost to disability worldwide [1]. BD is associated with poorer cognitive performance and quality of life, work impairment, mortality rates, and health care costs and utilisation [2, 3]. Evidence indicates that persistent cognitive deficits in BD are a critical determinant of clinical outcomes and functioning [4], and are an emerging treatment target in BD [5]. Specifically, trait‐related cognitive deficits have been observed across attention, verbal memory and learning, response inhibition, verbal fluency, and executive functions, with a prevalence of clinically relevant impairment in 50%–70% of individuals during periods of partial or full remission [6, 7, 8]. Importantly, this impairment may precede the illness onset [9], worsen as the illness progresses [10], impact negatively on functioning [11] and heighten the risk of psychiatric hospitalisations [12]. Nevertheless, the development of treatments targeting cognition in BD has proved challenging due to the limited understanding of the origins of cognitive impairment [5]. A better understanding of the factors contributing to cognitive impairment in BD would thus be beneficial for developing individualised interventions and prevention strategies.

Childhood maltreatment (CM), in the form of sexual, physical, and emotional abuse; physical and emotional neglect before the age of 18 [13, 14], is a key environmental factor contributing to the development of psychiatric conditions, including BD [15]. More than 50% of individuals with BD have a history of CM [16, 17]. Exposure to CM during periods of greater neurodevelopmental plasticity has been linked to lasting impairments in brain function and connectivity [18, 19]. CM exposure has also been strongly linked to disturbances in critical developmental skills (i.e., emotion regulation and social communication) and increased levels of chronic stress [20], which likely contribute to the development and progression of cognitive dysfunction across the life span [21]. Consistent with this, emerging evidence also suggests a significant association between CM and neuro‐ and socio‐cognitive impairments across various domains in individuals with different neuropsychiatric conditions [22, 23, 24], including BD [25, 26, 27].

To date, research exploring the relationship between CM and cognitive functioning in BD has produced mixed results. A recent systematic review found consistent evidence for CM being related to poorer cognitive functioning in mood disorders across global cognition and executive functions for euthymic patients, and psychomotor speed for in‐episode patients, with only mixed evidence for impaired verbal memory and executive function for in‐episode patients [25]. However, only four studies were conducted in BD populations, all except one were ‘euthymia’ studies, and social cognition was not explored. Another systematic review found an association between exposure to CM and worse global cognition, verbal and visual memory, processing speed and attention in mood disorders, and that specific trauma subtypes differentially associate with specific cognitive abilities [28]. However, the few studies conducted in BD were not enough to reach definitive conclusions. Only one previous meta‐analysis [24] examined the link between CM and neurocognition in adults with BD and found a significant association between CM and global cognition, and a small negative relationship between CM and working memory. However, this study was conducted in psychotic disorders, including BD with psychotic features (but not without psychotic features) and social cognition was not explored.

Furthermore, potential moderating (e.g., age, sex and BD type) or mediating factors (e.g., personality and biological stress) in the association between CM and cognitive functioning have seldom been studied. Taken together, the existing literature on the relationship between CM and cognitive function in BD is limited and inconsistent; no meta‐analytic studies have yet comprehensively evaluated the association between CM exposure and cognitive functioning in BD, and it remains unclear which specific cognitive domains are most affected, highlighting a significant gap in the research.

This systematic review and meta‐analysis sought to address these gaps by determining whether CM and its subtypes are associated with cognitive functioning, including global cognition and distinct cognitive domains (neurocognition, social cognition) in individuals with BD. These findings may help clarify factors contributing to the inconsistencies in the current literature. Understanding specific and global areas of dysfunction may help to shed light on mechanisms underlying the association. The review also explored potential moderators that may modify the strength and/or direction of associations between CM and cognitive functioning, and mediators that may explain these associations. Collectively, this information will enhance the development of mechanism‐specific theories and inform strategies for improving prediction, early identification, and targeted interventions.

## 2 Methods

### 2.1 Protocol

The review protocol was pre‐registered on PROSPERO (CRD42023468641). This review follows the Preferred Reporting Items for Systematic Reviews and Meta‐Analyses (PRISMA 2020) guideline [29] (see ST1 and ST2 in the Data S1), the Meta‐analysis of Observational Studies in Epidemiology (MOOSE) [30] (see ST3 in the Data S1), and the Enhancing the Quality and Transparency of Health Research (EQUATOR) [31] reporting guidelines. For a comprehensive glossary of terms used in this work, see SA1 in the Data S1.

### 2.2 Search Strategy and Selection Criteria

A systematic literature search using multiple Medical Subject Headings and keywords related to: (1) ‘child maltreatment’; (2) ‘bipolar disorder’ (3) ‘cognitive functioning’ OR ‘neurocognitive function’ OR ‘social cognition’ using the Boolean operator ‘AND’ (see the search strategy and terms appended in SA2 in the Data S1) was conducted in PubMed (Medline), PsycINFO, Embase, Web of Science (Core Collection), Cochrane, and PILOTS for inception on December 1st, 2023 and updated on June 24th, 2024. No language, age or date limits were applied. To identify additional eligible articles, the reference lists of the included articles were hand‐searched for additional studies. A snow‐balling approach was applied to identify additional studies meeting the inclusion criteria in the reference list of studies [32], and relevant studies already included in previous systematic reviews [28, 33] and meta‐analysis [26] were cross‐referenced manually (N.E.F.‐O.).

Titles and abstracts of articles in the initial search were independently screened by two reviewers (A.P.‐R., S.M.‐P.) (81.60% agreement); discrepancies were resolved with an independent reviewer (N.E.F.‐O.). After excluding irrelevant articles, full texts were independently assessed for eligibility by three reviewers (N.E.F.‐O., A.P.‐R. and S.M.‐P.); full‐text discrepancies were resolved through consensus. In the updated search, two reviewers (N.E.F.‐O. and A.P.‐R.) independently conducted title and abstract screening and full‐text assessment; discrepancies were resolved through consensus. The software Rayyan QCRI (https://rayyan.qcri.org/) was used to manage citations, remove duplicates and screening.

### 2.3 Inclusion and Exclusion Criteria

Included studies were empirical research articles published in a peer‐reviewed journal. Eligible studies reported quantitative associations between at least one CM subtype (exposure variable; that is, sexual, physical or emotional abuse; physical or emotional neglect) and at least one cognitive domain (outcome variable; global cognition/IQ, attention/processing speed, verbal memory/learning, working memory, executive functions/verbal fluency and social cognition) in individuals with BD, or included data that allowed correlations to be calculated, or provided these data on request (see the definition and operationalisation of exposure and outcome variables in SA3 in the Data S1). When more than one published study used the same subjects, the study with the larger sample size was chosen to maximise power.

Studies were excluded if they: (1) were reviews, clinical case studies, abstracts, conference proceedings, study protocols, letters to the editor not reporting original data, theoretical pieces or grey literature; (2) involved interventions and/or assessed treatment outcomes not providing baseline data.

According to the **PECOS** framework (Participants, Exposition, Comparators, Outcomes, Study Design), the method recommended for exploring associations between environmental and other exposures and health outcomes [34], studies were included if they: (1) **(P)** were conducted in individuals with BD, including BD type I or type II based on ICD [35] or DSM [36] criteria (see manual codes of BD diagnoses in ST4 in the Data S1); (2) **(E)** assessed the presence of CM before age 18, measured as overall (total) or specific CM subtypes; (3) **(C)** compared individuals with and without CM within the same sample of individuals with BD; (4) **(O)** evaluated cognitive functioning including neurocognitive function or social cognition with standard or validated instruments; **(S)** were cross‐sectional, cohort and case–control studies.

### 2.4 Study Outcomes

The selection of outcome domains was based on outcomes examined in the included studies, and similar categorisations and operationalisation used in previous meta‐analyses in the field [24, 26, 37].

After study selection, we categorised the study outcomes into: **(1) Global cognition and IQ:** general cognitive ability encompassing various cognitive domains such as memory, attention, executive functions, language, and visuospatial skills, and IQ presented as a composite score that summarises performance across diverse cognitive tasks [24, 38]; **(2) Attention and processing speed:** sustained and divided attention, and the capacity to process multiple sources of information simultaneously [39]; **(3) Verbal memory and learning:** recall or recognition of verbal stimuli and the process through which individuals acquire new information or skills [23, 40]; **(4) Working memory** [41]: cognitive system responsible for the temporary storage and manipulation of information necessary for complex cognitive tasks such as reasoning, comprehension, and learning to guide decision‐making and behaviour; **(5) Executive functions and verbal fluency** [42]: set of higher‐order cognitive processes that enable goal‐directed behaviour, problem‐solving, and adaptive responses to novel or complex situations, including planning, inhibitory control, task switching, and the capacity to generate words rapidly and efficiently within specific constraints, reflecting lexical retrieval, language production, and cognitive flexibility [23]; and **(6) Social cognition** (i.e., including emotion perception, theory of mind) [43, 44]: processes to perceive, infer, and decode social information and abilities to ‘make sense of others' behavior’ [45], including recognition and managing emotions, and the ability to reason about mental states and understand intentions, dispositions, emotions, and beliefs of oneself and others.

Appendix SA3 in the Data S1 provides a complete definition and operationalisation of each outcome domain and ST5 provides a complete overview of assessments (neuropsychological tests) of each outcome domain.

### 2.5 Data Extraction and Quality Assessment

Data from eligible studies were extracted and tracked in Microsoft Excel by three independent reviewers (A.P.‐R., R.L.‐E. and S.M.‐P.) using a structured template and coding form; discrepancies were discussed with an independent reviewer (N.E.F.‐O.) and resolved through consensus.

Descriptive variables extracted comprised demographics, illness characteristics and measurement instruments for BD, CM and cognitive functioning (see a detailed description of the extracted variables in SA4 in the Data S1). In each study, authors' (of the original papers) criteria for classifying neuropsychological tests into cognitive domains were respected.

Correlation coefficients (*r*) were extracted as measures of effect size, or data from which correlations could be calculated. In the case where no overall CM effect was reported, only the effects of specific subtypes of CM were extracted. For longitudinal studies, data indicating associations at baseline were extracted. Corresponding authors were contacted by email twice to retrieve additional information if necessary.

The quality assessment was independently conducted by two independent reviewers (N.E.F.‐O. and R.L.‐E.) (89.38% agreement) using an adapted version of the Newcastle Ottawa Scale (NOS) [46] for non‐randomised studies as employed in previous meta‐analyses [26, 37], which contains additional items to assess sample size, confounders and statistical tests, as recommended by the Cochrane Handbook [47] (see ST6 in the Data S1). Discrepancies were resolved through consensus.

### 2.6 Statistical Analysis

Random‐effect meta‐analyses [48] when a minimum of three studies were available were conducted. If the number of available effect sizes did not allow random effects meta‐analysis, study findings were summarised and appraised qualitatively.

For those studies not reporting correlation coefficients, information was transformed from available statistics (e.g., mean and standard deviations between groups comparisons, regression coefficients and odds ratios), as per procedures used in previous meta‐analyses [26, 37] using established formulas with the ‘Practical Meta‐Analysis Effect Size Calculator’ [49]. Pearson correlation coefficients (effect sizes) were Fisher's *Z* transformed to stabilise the variance and calculate reliable confidence intervals (CIs) and back transformed after pooling to allow for clearer interpretation, as done in previous research [26, 37]. Thus, all pooled effects are reported as correlation coefficients.

Heterogeneity was assessed using Cochran's *Q*‐test and *I* ^2^ statistics, with substancial heterogeneity being indicated by *I* ^2^ ≥ 50% [50], with 25%, 50% and 75% defining thresholds for low, moderate and high heterogeneity [50]. Alongside the 95% CIs and the mean pooled effect provided, the prediction intervals estimating the extent to which effect sizes vary across studies [51] were displayed as part of the forest plots (marked in red).

One‐study‐removed sensitivity analyses were conducted to determine whether a particular study or a set of studies were contributing to potential heterogeneity. In addition, a series of random‐effect meta‐regressions [52] were conducted on the following pre‐selected variables: mean sample age, percentage of male individuals, percentage of BD type I, sample size and study quality (NOS rating). Other evidence of confounders and effect moderators and mediators examined in the included studies on associations between CM and cognitive functioning was narratively synthesised [53].

For associations including at least 10 studies, publication bias was examined, funnel plots were visually inspected, and the intercept Egger's test was used to numerically explore the risk of publication bias (i.e., Egger's test *p* value < 0.05) [47, 54]. Where indications of publication bias were found, corrected effect sizes using the Duval and Tweedie's trim‐and‐fill method were additionally reported to correct for significant risk of bias [55].

Statistical significance was evaluated two‐sided at the 5% threshold (two tailed). Interpretation of correlation coefficients was based on pre‐defined cut‐offs as follows: *r* values between 0 and 0.3 indicate small, values between 0.3 and 0.7 indicate moderate, and values above 0.7 indicate strong associations [56].

All quantitative analyses were performed using Comprehensive Meta‐Analysis v4.0 (CMA, version 4‐meta‐analysis.com) [57], and the figure illustrating the meta‐analytic synthesis was created using the *ggplot2* package using R version 4.1.2 [58].

## 3 Results

### 3.1 Study Selection

**FIGURE 1:**
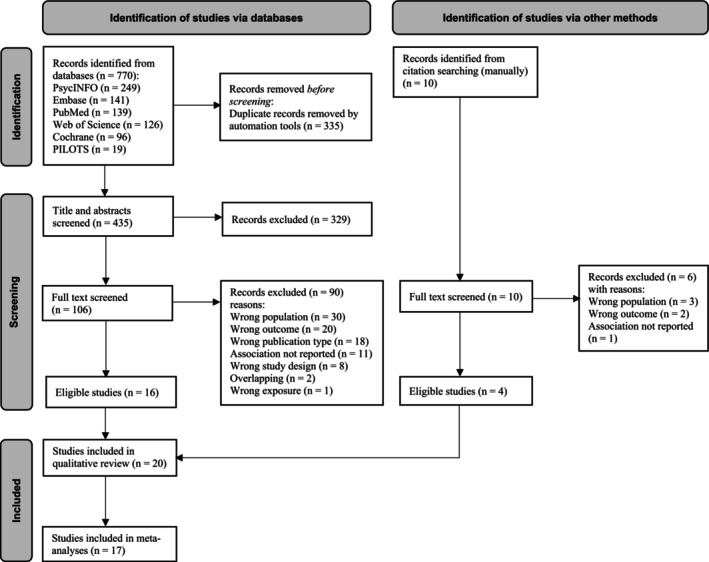
PRISMA 2020 flowchart outlining the study selection process.

From 780 identified records (770 through data bases and 10 studies through manual searches), 116 were full‐text screened, and 20 studies were included in the qualitative synthesis, of which 17 were included in the quantitative synthesis, contributing to 152 effect sizes pooled in meta‐analyses (see the process of study selection in detail in Figure 1, the full list of included studies in SA5 and excluded studies with reasons in SA6 in the Data S1).

### 3.2 Characteristics of the Included Studies

The 20 included studies were published between 2010 and 2024 and were conducted in Europe (*n* = 5), South America (*n* = 5), North America (*n* = 5), Turkey (*n* = 3), Asia (*n* = 1), and Australia (*n* = 1). Most of the included studies were cross‐sectional (84.2%) except for three studies with a longitudinal design [59, 60, 61].

The total sample of the included studies compromised 2457 individuals with BD, sample size [range = 38–405], of which 41.3% were males. The mean age was 39.5, SD = 9.7, [range = 12.24–50.94] years. The mean education level was 13.4 (SD = 2.2) years (secondary education completed). Eight studies [23, 59, 60, 61, 62, 63, 64, 65, 66] provided information on premorbid IQ (mean = 105.2, SD = 7.8, [range = 86.9–112.2]). More than 80% of the samples for 16 of the included studies fulfilled criteria for BD type I (vs. type II); in one study [67], the samples were mixed (type I and type II) [67], and in three studies [68, 69, 70] the BD type was not reported. One of the included studies [59] was conducted in patients with first episode of mania [59]; one study was conducted in youths [range = 12–22 years] [61]. Most of the studies (*n* = 14) were conducted in patients in euthymic state; one of these studies [59] included patients who recently recovered from a first episode of mania [59]. Two studies [61, 67] included mixed samples involving patients in euthymic and in‐episode states [61, 67], and four studies [44, 60, 69, 71] did not report the status of patients at the time of assessment [44, 60, 69, 71].

The mean age of illness onset (mean = 21.12, SD = 5.4 years) was reported in nine studies [44, 59, 60, 61, 62, 68, 72, 73, 74]. Six studies [23, 62, 64, 65, 68, 74] reported the duration of illness (mean = 16.7, SD = 5.8 years) [23, 62, 64, 65, 68, 74]. Eight studies [23, 59, 62, 63, 66, 68, 72, 74] reported the number of manic episodes (mean = 3.80, SD = 2.87), and depressive episodes (Mean = 5.3, SD = 5.5) [23, 59, 62, 63, 66, 68, 72, 74]. Two studies [23, 74] reported the mean (14.0, SD = 11.1) of the total number of episodes, including both depressive and manic episodes.

Seven studies [63, 64, 68, 71, 72, 74, 75] reported the mean (0.58, SD = 0.35) of the total number of suicide attempts. Two studies [60, 62] reported the percentage of patients with a history of suicide ideation and/or attempts. Jiménez et al. reported 34.56% of patients had suicidal ideation while 27.50% attempted suicide [62]. Ehrlich et al. reported 31.22% of patients with high trauma while 24.15% of patients with low trauma had a history of a suicide attempt [60]. Seven studies [60, 62, 63, 68, 73, 74, 75] reported the mean of the total number of hospitalisations (3.00, SD = 0.87). Five studies [23, 62, 64, 66, 69] reported the percentage of patients (49.7%) undergoing pharmacological treatment with lithium [23, 62, 64, 66, 69]. None of the studies reported enrolment to any cognitive or other psychosocial interventions.

All the included studies used retrospective assessment of CM. The Childhood Maltreatment Questionnaire (CTQ) short‐form (28 items) [76] was used in 18 studies, including shortened (25 items) or translated versions. For seven studies [43, 44, 60, 61, 63, 65, 70] CM exposure was dichotomised (yes/no, or high/low or minimal [43, 44, 60, 61, 63, 65, 70]), and for 13 studies [23, 59, 62, 64, 66, 67, 68, 69, 71, 72, 73, 74, 75] it was measured on a continuous scale (cumulative or severity total score).

Most of the studies used semi/structured interview (*n* = 13), six studies used clinical rating (DSM criteria), and one study [59] used both semi/structured and clinical rating for diagnosis evaluation. The SCID‐Structured Clinical Interview (*n* = 7) for DSM‐IV (*n* = 5) and DSM‐5 (*n* = 2) was the most used diagnostic instrument. Three studies [70, 73, 75] did not specify the diagnostic instrument. See further details of sociodemographic and clinical characteristics of the included studies in Table 1.

**TABLE 1:** Sociodemographic and clinical characteristics, and main findings of the included studies investigating the association between CM and cognitive functioning in BD.

Authors (year)	Country/region	*n* (total BD)	BD Diagnosis (% type I)	Mood status	Instrument for diagnosis/criteria	Sex (% male)	Mean age (in years)	Instrument to assess CM	Type of CM	Measures of Cognitive domain(s)	Main findings	Confounders
Aas et al. (2012)	Norway/Europe	167	64	18.4% Bipolar II with psychotic symptoms during depression	SCID‐I/DSM‐IV	NA	NA	28‐Items‐CTQ Norwegian version	EA, PA, SA, EN, PN	IQ (performance, verbal): WASI (Block design, Matrix reasoning, Similarities, Vocabulary) Verbal memory: CVLT Working memory: LNS, WAIS‐III (Digit span forwards/backwards) Verbal fluency: Verbal fluency, Letter fluency	CM (PA, SA and PN) was associated with a reduction in cognitive function across cognitive domains in patients with BD, in particular working memory and executive function as well as general cognition	Age, sex
Arat‐Çelik et al. (2023)	Turkey/Europe‐Asia	41	NA	Euthymia (for a minimum of 4 months)	SCID‐5/DSM‐5	34.15	34.80	28‐Items‐CTQ Turkish version	EA, PA, SA, EN, PN	Global cognition: RAVLT, DST, SCWT, ACT, Digit Symbol Test, COWAT, Animal naming, TMT, VCT Attention and processing speed: TMT‐A‐B, Digit Symbol Test Verbal memory: RAVLT, WMS‐R, VCT Working memory: WAIS‐R (Digit Span), ACT Executive functions (response inhibition): SCWT (Interference score) and verbal fluency: COWAT, Animal Naming test	Significant negative correlations between CM (PN, SA) and neurocognitive domains like processing speed, verbal fluency, working memory, and the global cognitive factor were observed	Age, sex, education
Bücker et al. (2013)	Canada/North America	64	100	Euthymia (recently recovered from a first episode of mania	MINI/DSM‐IV‐TR	NA	22.89	28‐Items‐CTQ	Overall CM	IQ (general, verbal, motor): K‐BIT Vocabulary, Matrices Attention and processing speed: CANTAB (RVIP), CVLT trial 1, TMT‐A, SCWT (Word‐Colour score), FAS, Letter fluency Verbal memory: CVLT Non‐verbal memory: CANTAB (paired associate learning) Working memory: CANTAB (SWM), WMS‐III (LNS) Executive functions: SCWT (Interference score), TMT‐B, CANTAB (IED/stockings problems)	CM was associated with poorer cognitive performance on cognitive measures of IQ, auditory attention, verbal and working memory in BD patients who recently recovered from a first episode of mania	Age, sex, education
Ehrlich et al. (2023)	USA/North America	405	68.64	NA	DIGS	31.85	39.29	28‐Items‐CTQ	Overall CM	Attention and processing speed: TMT‐A‐B, Digit Symbol coding Test, SCWT (Word‐Colour score) Verbal memory: CVLT Visual memory: ROCF Motor function: Purdue Pegboard Executive functions and verbal fluency (conceptual reasoning and set‐shifting, processing speed with inference resolution, verbal fluency with processing speed, inhibitory control): WCST, PGNG, TMT‐B, WAIS‐III (Digit Symbol Coding Test), SCWT (Interference score), COWAT (FAS) Social cognition: Emotion Perception Test, Test of Facial Recognition	Comparing the high and low trauma BD groups, high trauma was related to lower auditory and visual memory factors. As compared to HCs, the BD high trauma group had lower scores on six of eight cognitive factors, while the BD low trauma group had lower scores on four of eight cognitive factors. No differences were found in conceptual reasoning and set‐shifting or emotion processing among groups	Age, sex, education, depression severity, estimated IQ
Hsieh et al. (2021)	Taiwan/Asia	38	NA	NA	DSM‐IV	31.58	37.24	28‐Items‐CTQ	EN, PN	Executive functions (cognitive flexibility): WCST	The level of neglect exposure was negatively correlated with WCST performance; and the left‐caudate‐seed functional connectivity to the fronto‐parietal network was positively correlated with WCST performance in BD	Age, education, mood symptoms
Jiménez et al. (2017)	Spain/Europe	113	71.68	Euthymia	SCID/DSM‐IV‐TR	40.71	47.79	28‐Items‐CTQ Spanish version	Overall CM, EA, PA, SA, EN, PN	Attention and processing speed: CPT‐II WAIS‐III (symbol/digit‐symbol) COWAT (FAS), TMT‐A Working memory: WAIS‐III (Arithmetic, digit/LNS) Verbal memory and learning: CVLT Visual memory: ROCF Executive functions (response inhibition, set shifting, planning): TMT‐B, SCWT (Interference score), WCST Social cognition (emotion intelligence): MSCEIT‐2.0	CTQ scores, especially on the PN subscale, were associated with worse cognitive performance in attention, working memory, verbal memory and learning in adulthood in BD	Education, estimated IQ, depressive symptoms
*Larsen et al. (2019)	USA/North America	62	85	Euthymia	SCID‐5/DSM‐5	38.71	38.30	28‐Items‐CTQ	Overall CM, EA, PA, SA, EN, PN	Moral decision‐making: Moral dilemma task (personal‐intentional, personal‐unintentional, impersonal‐intentional, impersonal‐unintentional types)	Higher ratings of PN were significantly associated with higher ratings of acceptability (a utilitarian tendency) across dilemma types	Diagnosis, sex, race, age, premorbid IQ, education, mood ratings
Lima et al. (2017)	Brazil/South America	117	67	NA	MINI Plus 5.0/DSM‐IV	33.33	43.60	28‐Items‐CTQ Brazilian version	EA, PA, SA, EN, PN	Executive functions (inhibition control, non‐planning): BIS‐11	Inhibitory control and EA were related, conjointly, to a greater likelihood of having a history of more than one suicide attempt	Depression and mania ratings
Martins et al. (2019)	Brazil/South America	72	85.92	Euthymia	SCID/DSM‐IV	33.33	45.89	28‐Items‐CTQ Brazilian version	Overall CM, EA, PA, SA, EN, PN	IQ (global): WASI	PA, EA and EN were significantly associated with a decrease in estimated IQ in patients with BD, affecting overall cognitive development	Family history of severe mental disorders, age at diagnosis, psychotic symptoms during the first episode
Miskowiak et al. (2023)	Denmark/Europe	345	36.50	Euthymia (full or partial remission)	ICD‐10	37.10	33.47	28‐Items‐CTQ	Overall CM, EA, PA, SA, EN, PN	Global cognition: global cognitive composite of individual cognitive domains IQ: DART Attention: TMT‐A, RBANS (Digit span forward), CANTAB (RVP) Psychomotor speed: SCIP (psychomotor speed task), RBANS (coding simple reaction time) Verbal memory and learning: SCIP (verbal learning and delayed recall), RAVLT Working memory: SCIP (working memory task), WAIS‐III (LNS), SWM Executive functions and verbal fluency: TMT‐B, CANTAB‐OTS (flexibility, spatial planning), SCIP (verbal fluency)	Patients with BD showed cognitive impairments across memory, attention and executive functions despite being in partial or full remission and had higher levels of CM than HCs. Higher levels of CM correlated with impairments across almost all cognitive domains in both BD and HCs. Associations between CM and poorer working memory, prevailed after adjusting for clinical and demographical variables. Diagnosis of BD and estimated verbal IQ did not moderate these associations. Working memory impairments were related particularly to PA and EA	Diagnostic group, (BD type I/II), sex, age, verbal IQ, mood symptoms
Morán‐Kneer et al. (2022)	Chile/South America	76	100	Euthymia (for at least 3 months)	DSM‐IV‐TR	34.21	49.50	28‐Items‐CTQ Chilean‐ Spanish version	Overall CM, EA, PA, EN, PN	Social cognition (ToM): Hinting task test	Higher scores on attachment anxiety were positively associated with CM and better social cognition performance	NA
*Mowlds et al. (2010)	Northern Ireland/Europe	52	NA	Euthymia (current relapse of disorder as exclusion criteria)	DSM‐IV	40.38	50.94	28‐Items‐CTQ THQ	Overall CM, EA, PA, SA, EN, PN	Autobiographical memory (episodic and semantic memory): AMT	Whilst CM predicted current inter‐episode depressive mood, CM was not predictive of BD severity or autobiographical memory specificity	NA
*Oymak‐Yenilmez et al. (2021)	Turkey/Europe‐Asia	85	75.29	Euthymia (in remission)	SCID‐I/DSM‐IV‐TR	45.88	36.56	28‐Items‐CTQ Turkish version	Overall CM	Metacognitions: MCQ‐30	Metacognitions impairments were predicted by SA in BD	Age, education, age of BD onset, number of episodes, number of hospitalisations
Quidé et al. (2018)	Australia/Oceania	84	100	NA	ICD‐10	35.71	37.47	25‐Items‐CTQ	Overall CM	Attention: RBANS‐Attention Verbal memory and learning: RBANS‐immediate and delayed memory Working memory: WAIS‐R‐III (LNS) Verbal fluency: COWAT Social cognition (facial emotion recognition, ToM): FEEST, TASIT	CM‐exposed individuals showed deficits specifically in social cognition, but not general cognitive abilities especially in the context of higher schizotypy levels	Sex, age
Rios et al. (2020)	Chile/South America	117	100	Euthymia (for at least 3 months)	DSM‐IV‐TR	34.00	45.50	28‐Items‐CTQ Spanish version	EA, PA	Orientation, Attention, Memory, Language, Visuospatial abilities: ACER‐R Verbal fluency: ACER‐R Social cognition (emotion recognition, ToM, empathic distress): TASIT, IRI	CA was associated with specific disturbances in social cognition tasks	NA
Rios et al. (2023)	Chile/South America	101	100	Euthymia	DSM‐IV‐TR	39.61	47.30	28‐Items‐CTQ Spanish version	Abuse (EA, PA, SA) EA, PA, SA EA, SA PA, SA EA, PA	Social cognition (emotion recognition, ToM): TASIT	BD‐Type I patients who had been victims of PA and EA in childhood and were carriers of the GG genotype at *OXTR rs53576* displayed greater social cognition alterations, specifically in emotion recognition	NA
Russo et al. (2015)	USA/North America	75	70.70	Euthymia (affectively stable)	SCID/DSM‐IV	68.00	47.10	28‐Items‐CTQ	PA, EN, PN	Social cognition (emotion recognition): CANTAB (ERT)	BD patients with a positive childhood history of EN perform worse than those without such a history in recognising anger	Age, duration of illness
Takim et al. (2024)	Turkey/Europe‐Asia	50	100	Euthymia (in remission)	DSM‐5	52.00	36.96	28‐Items‐CTQ	Overall CM, EA, PA, SA, EN, PN	Social cognition (ToM): DEZIKO	A negative correlation was identified between PN and detecting social errors as measured by the DEZIKO faux pas score. BD patients had lower ToM abilities despite being in remission. A relationship between ToM, alexithymia, and CM in BD was found	Age, gender, educational status
Vaughn‐Coaxum et al. (2021)	USA/North America	198	55.60	Participants spent 50% of time in euthymia, 36% of time with sub‐threshold depressive symptoms, and 11% of time with full‐threshold depressive symptoms	KSADS‐ PL/DSM‐IV	56.10	16.58	KSADS LEC	PA, SA	General IQ: WASI Attention: CANTAB (RVIP) Executive functions (inhibitory control): CANTAB (AGN)	In the context of lower sustained attention, maltreatment exposure was associated with higher depression symptom severity during childhood, but not late adolescence. There was no association between maltreatment and symptom severity in the context of higher sustained attention, and no association between attention and depression symptom severity for non‐maltreated youths	Comorbid ADHD, age of BD onset, family SES, sex, general IQ score
Vreeker et al. (2017)	The Netherlands/Europe	195	100	Euthymia (no mood episode in the 4 weeks prior to the interview)	SCID‐I/DSM‐IV	49.20	48.00	28‐Items‐CTQ	Overall CM	IQ (General knowledge, processing speed, working memory, visuospatial capacities): WAIS‐III (Information, Digit Symbol coding, Arithmetic, Block design)	CM was negatively correlated with IQ. CM and use of lithium and antipsychotic medication did not affect the relationship between brain volumes and IQ. However, current lithium use was related to lower IQ in patients with BD	Current lithium and antipsychotic medication use

*Note:* * Studies not included in meta‐analysis but fulfilling inclusion criteria and included in the systematic review.

Abbreviations: ACER/*R* = Addenbrooke's Cognitive Examination‐Revised Chilean; ACT = Turkish version of the Auditory Consonant Trigrams Test; ADHD = Attention deficit hyperactivity disorder; AGN = CANTAB's Affective Cognitive Go/No go Task; AMT = Autobiographical Memory Test; BD = Bipolar Disorder; BIS‐11 = Barrat Impulsiveness Scale; CA = Child abuse; CANTAB = Cambridge Neuropsychological Test Automated Battery; CM = Childhood Maltreatment; COWAT = Controlled Oral Word Association Test; CPT‐II = Conner's Continuous Performance Test II; CTQ = Childhood Trauma Questionnaire; CVLT = California Verbal Learning Test; DART = National Adult Reading Task, Danish translation; DEZIKO = Dokuz Eylul Theory of Mind Index; DIGS = Diagnostic Interview for Genetic Studies; DSM‐5 = Diagnostic and Statistical Manual of Mental Disorders Fifth Edition; DSM‐IV = Diagnostic and Statistical Manual of Mental Disorders Fourth Edition; DSM‐IV‐TR = Diagnostic and Statistical Manual of Mental Disorders Fourth Edition Revision; EA = Emotional abuse; EN = Emotional neglect; ERT = CANTAB's Emotion Recognition Task; FAS = Word Fluency of Controlled Oral Word Association Test; FEEST = Facial Expressions of Emotion Stimuli and Tests; HCs = healthy controls; ICD‐10 = International Classification of Diseases 10th Revision; IED = CANTAB's Intra‐/extra‐dimensional set‐shifting task; IQ = Intellectual Quotient; IRI = Interpersonal Reactivity Index; K‐BIT = Kaufman Brief Intelligence Test; KSADS = Kiddie Schedule for Affective Disorders and Schizophrenia; KSADS‐PL = Kiddie Schedule for Affective Disorders and Schizophrenia Present and Lifetime Version; LEC = Life Events Checklist; LNS = Letter Number Sequencing; MCQ‐30 = Metacognitive Questionnaire‐30; MINI = Mini International Neuropsychiatric Interview; MSCEIT 2.0 = Mayer‐Salovey‐Caruso Emotional Intelligence Test Version 2.0; NA = Not available; NOS = Newcastle‐Ottawa Scale (total score); OTS = CANTAB's One Touch Stockings of Cambridge test; OXTR = Oxytocin Receptor Gene; PA = Physical Abuse; PGNG = Parametric Go/No‐Go; PN = Physical Neglect; RAVLT = Rey Auditory Verbal Learning Test; RBANS = Repeatable Battery for the Assessment of Neuropsychological Status; ROCF = Rey–Osterrieth complex figure Test; RVIP = CANTAB's Rapid Visual Information Processing; RVP = Rapid Visual Processing Task; SA = Sexual Abuse; SCID = Structured Clinical Interview; SCID = Structured Clinical Interview for DSM; SCIP = Screen for Cognitive Impairment in Psychiatry; SCWT = Stroop Colour‐Word Test; SES = Socioeconomic Status; SRM = CANTAB's Spatial Recognition Memory Test; SWM = CANTAB's Spatial Working Memory Test; TASIT = Awareness of Social Interference Test; THQ = Trauma History Questionnaire; TMT‐A = Trail Making Test Version A; TMT‐B = Trail Making Test Version B; ToM = Theory of Mind; VCT = WMS‐R's Visual Copying Test; WAIS‐III = Wechsler Adult Intelligence Scale Third Edition; WAIS‐*R* = Wechsler Adult Intelligence Scale‐Revised; WASI = Wechsler Abbreviated Scale of Intelligence; WCST = Wisconsin Card Sort Test; WMS‐III = Wechsler Memory Scale Third Edition; WMS‐*R* = Wechsler Memory Scale‐Revised.

### 3.3 Study Quality

The mean quality rating [range = 0–8] of the included studies was 6.2 [range = 4–8]. Overall, two (10%) studies were rated as ‘poor’ (NOS score = 4), two (10%) studies were rated as ‘fair’ (NOS score = 5), eight (40%) studies were rated as ‘good’ (NOS score = 6), and eight (40%) studies received a rating considered as ‘high’ (NOS score > 6) (see further details of the study quality assessment in ST6 in the Data S1).

### 3.4 Qualitative Synthesis and Meta‐Analytic Results of Associations Between CM and Cognitive Functioning in BD

Across samples of the included studies, over half of participants of the included studies had at least one type of CM experience during childhood. Among the 20 studies reviewed, overall CM was examined in 12 articles [23, 44, 59, 60, 62, 64, 66, 74, 75]; 12 studies explored emotional abuse [23, 43, 62, 63, 64, 67, 68, 70, 71, 73, 74, 75]; 14 articles explored physical abuse [23, 43, 61, 62, 63, 64, 65, 67, 68, 70, 71, 73, 74, 75], 11 studies explored sexual abuse [23, 61, 63, 64, 67, 68, 70, 71, 73, 74], 12 studies explored emotional and physical neglect [23, 62, 63, 64, 65, 67, 68, 69, 70, 71, 74, 75]. No studies investigated domestic violence or bullying exposure.

Across the included studies, a variety of neuropsychological tests were used to measure global cognitive function and IQ (*k* = 19), and five cognitive domains: attention and processing speed (*k* = 22), verbal learning and memory (*k* = 25), working memory (*k* = 24), executive functions and verbal fluency (*k* = 35), and social cognition (*k* = 27).

**FIGURE 2:**
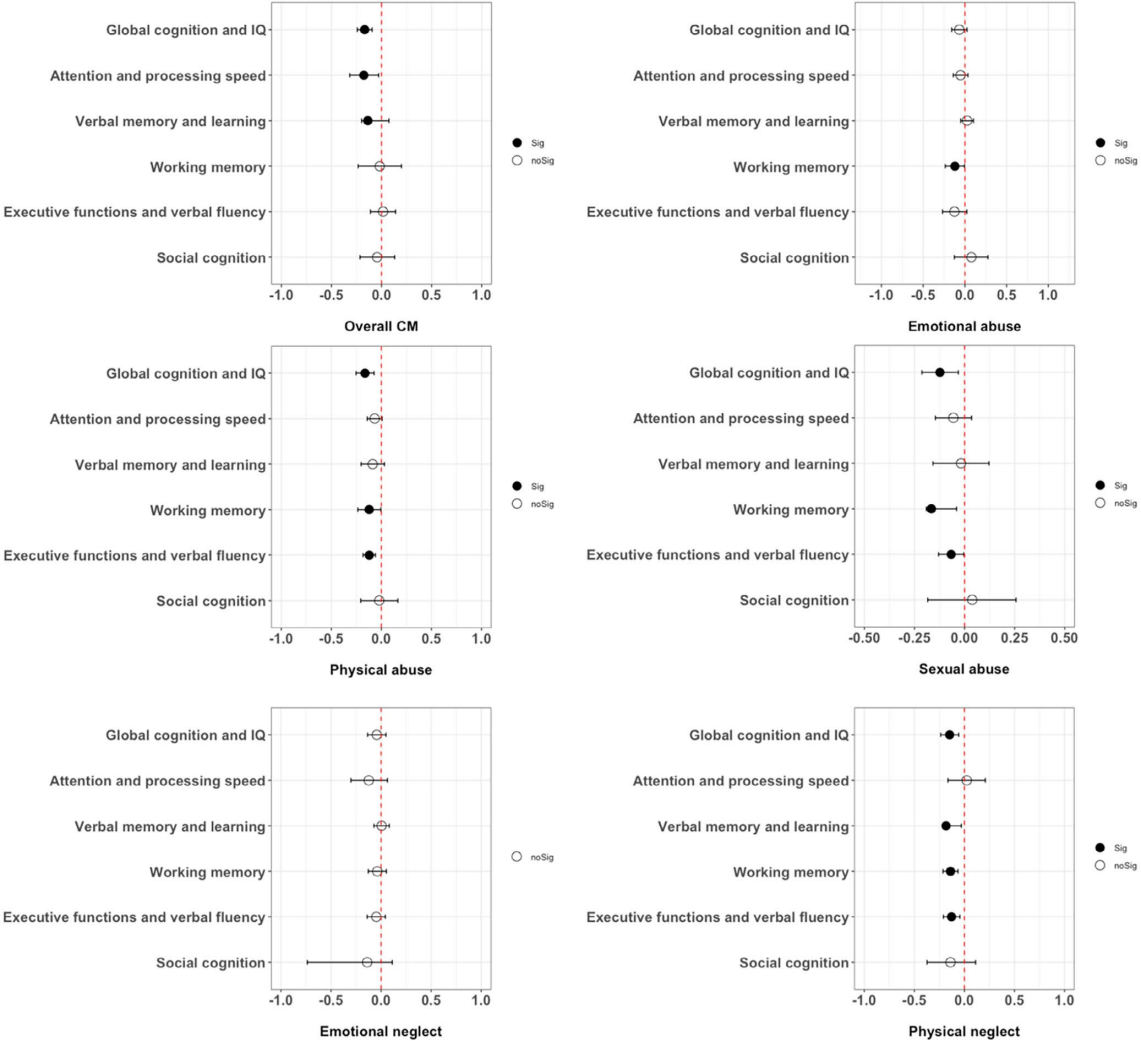
Overall results of the meta‐analytic synthesis.

Separate meta‐analyses with random‐effects estimates were calculated to quantify associations between global cognition/IQ and cognitive domains and CM separated by overall and subtypes. The main results are presented in Table 2 and illustrated in Figure 2. Forest plots of each analysis can be found in SA7 in the Data S1.

**TABLE 2:** Meta‐analyses of associations between CM and cognitive outcomes in BD.

Childhood maltreatment (CM) total/subtypes	Number of studies (n), effect sizes *(k)*	Pooled sample size	Correlation coefficient			Heterogeneity		Prediction intervals
			*r*	95% CI	*p‐*value	*I* ^*2*^ (%)	Q test *p‐*value	
	**Global cognition and IQ**							
Overall CM	4 (*4*)	676	−0.169	−0.242; −0.094	< 0.001	0	—	—
Emotional abuse	3 (*3*)	458	−0.067	−0.158; 0.026	0.157	0	—	—
Physical abuse	3 (*3*)	458	−0.164	−0.253; −0.073	< 0.001	0	—	—
Sexual abuse	3 (*3*)	458	−0.123	−0.213; −0.031	0.009	0	—	—
Emotional neglect	3 (*3*)	458	−0.043	−0.135; 0.049	0.358	0	—	—
Physical neglect	3 (*3*)	458	−0.148	−0.237; −0.057	0.002	0	—	—
	**Neurocognition**							
	**Attention and processing speed**							
Overall CM	5 (*5*)	1011	−0.177	−0.317; −0.029	0.019	79	0.001	−0.607; 0.333
Emotional abuse	3 (*3*)	499	−0.052	−0.139; 0.037	0.251	0	—	—
Physical abuse	4 (*4*)	697	−0.067	−0.141; 0.008	0.079	0	—	—
Sexual abuse	4 (*4*)	697	−0.056	−0.146; 0.035	0.227	23	0.270	−0.325; 0.221
Emotional neglect	3 (*3*)	499	−0.123	−0.301; 0.064	0.196	66	0.052	−0.976; 0.960
Physical neglect	3 (*3*)	499	0.023	−0.165; 0.209	0.816	67	0.049	−0.969; 0.972
	**Verbal memory and learning**							
Overall CM	5 (*5*)	1011	−0.137	−0.199; −0.074	< 0.001	3	0.392	−0.243; −0.028
Emotional abuse	4 (*4*)	666	0.026	−0.050; 0.103	0.498	0	—	—
Physical abuse	4 (*4*)	666	−0.087	−0.203; 0.031	0.147	48	0.123	−0.482; 0.337
Sexual abuse	4 (*4*)	666	−0.018	−0.158; 0.122	0.799	63	0.044	−0.528; 0.501
Emotional neglect	4 (*4*)	666	0.005	−0.071; 0.082	0.895	0	—	—
Physical neglect	4 (*4*)	666	−0.184	−0.184; −0.031	0.006	1	0.385	−0.277; 0.066
	**Working memory**							
Overall CM	4 (*4*)	606	−0.018	−0.233; 0.199	0.875	83	0.001	−0.766; 0.751
Emotional abuse	4 (*4*)	666	−0.120	−0.194; −0.044	0.002	0	—	—
Physical abuse	4 (*4*)	666	−0.122	−0.235; −0.005	0.042	48	0.125	−0.506; 0.303
Sexual abuse	4 (*4*)	666	−0.166	−0.191; −0.040	0.003	0	—	—
Emotional neglect	4 (*4*)	666	−0.038	−0.128; 0.053	0.417	20	0.292	−0.298; 0.228
Physical neglect	4 (*4*)	666	−0.139	−0.214; −0.064	< 0.001	0	—	—
	**Executive functions and verbal fluency**							
Overall CM	6 (*6*)	1011	0.015	−0.111; 0.140	0.820	70	0.010	−0.385; 0.409
Emotional abuse	5 (*5*)	783	−0.125	−0.268; 0.024	0.100	73	0.005	−0.562; 0.367
Physical abuse	6 (*6*)	981	−0.121	−0.183; −0.059	< 0.001	0	—	—
Sexual abuse	6 (*6*)	981	−0.067	−0.130; −0.004	0.037	0	—	—
Emotional neglect	6 (*6*)	821	−0.048	−0.139; 0.043	0.297	32	0.197	−0.259; 0.167
Physical neglect	6 (*6*)	821	−0.129	−0.210; −0.045	0.003	22	0.265	−0.302; 0.052
	**Social cognition**							
	**Emotion perception and theory of mind**							
Overall CM	5 (*5*)	728	−0.044	−0.215; 0.131	0.625	77	0.002	−0.576; 0.515
Emotional abuse	5 (*5*)	457	0.078	−0.126; 0.277	0.453	79	0.043	−0.578; 0.673
Physical abuse	6 (*6*)	532	−0.021	−0.206; 0.165	0.824	78	0.042	−0.573; 0.544
Sexual abuse	3 (*3*)	264	0.038	−0.184; 0.257	0.737	68	0.044	−0.986; 0.988
Emotional neglect	4 (*4*)	314	−0.120	−0.381; 0.158	0.397	83	< 0.001	−0.884; 0.819
Physical neglect	4 (*4*)	314	−0.139	−0.373; 0.112	0.276	79	0.002	−0.853; 0.756

*Note:* significance *p* < 0.05.

For a description of the main results of other cognitive domains evaluated among the included studies but not providing sufficient data for meta‐analysis, see Table 1 and SA8 in the Data S1.

#### 3.4.1 Global Cognition and IQ

Studies evaluated global (or general) cognition reporting a global cognitive composite score of individual cognitive domains (*n* = 2) [23, 68] and/or reported a general IQ (*n* = 6) [23, 59, 61, 64, 66, 67] derived from the Wechsler abbreviated scale of intelligence (WASI), involving Block Design and Matrix Reasoning tasks to measure perception and visuo‐spatial abilities (performance subscale), and Similarities and Vocabulary tasks to measure verbal abilities (verbal subscale) (*n* = 1) [67], the global score of the WASI Second edition (WASI‐II) [77] (*n* = 3) [60, 61, 64], and the Digit Symbol Coding, Block Design, Arithmetic and Information subtests of the Wechsler Adult Intelligence Scale Third edition (WAIS‐III) [78] (*n* = 1) [66]. Other studies reported verbal IQ using the Adult Reading Task, Danish translation (DART), and the vocabulary subtest of the WAIS‐III [78] (*n* = 2) [23, 62], or both general and verbal IQ estimated with the Kaufman Brief Intelligence Test (K‐BIT) [79] (*n* = 1) [59].

Six studies found significant associations between CM and impaired global cognition [23, 68] and IQ [59, 64, 66, 67]. One of these studies [23] reported an association between CM and global cognition in patients despite being in partial or full remission, or type of BD.

Random‐effect meta‐analyses showed that most of the CM subtypes, including overall CM, physical and sexual abuse and physical neglect, were negatively associated with global cognition and IQ (*k* = 3 to 4; *r* = −0.12 to −0.17, *p* = 0.009 to < 0.001).

#### 3.4.2 Attention and Processing Speed

Studies evaluated attention and processing speed [23, 44, 59, 61, 62, 68] using the Trail Making Test forms A and B (TMT‐A‐B) [80] and the Stroop Colour and Word Test (SCWT) [81] (Word‐Colour score), the computerised version of the Conner's Continuous Performance Test (CPT‐II) [82], digit/symbol tasks of the WAIS‐III (*n* = 3) [59, 62, 68], the Rapid Visual Information Processing (RVIP) test of the Cambridge Neuropsychological Test Automated Battery (CANTAB) [61] (*n* = 1) [61], and the Digit span forward of the RBNAS [83] (*n* = 2) [23, 60].

Overall, studies found significant associations between CM and impaired auditory and sustained attention and processing speed. Conversely, one study [60] found no differences between CM‐exposed and non‐exposed individuals with BD.

Random‐effect meta‐analyses showed that overall CM (but none of the CM subtypes) was negatively associated with attention and processing speed (*k* = 5; *r* = −0.18, *p* = 0.019).

#### 3.4.3 Verbal Memory and Learning

Studies examined associations between CM and verbal memory/learning [23, 43, 44, 59, 60, 62, 67, 68] using the California Verbal Learning Test (CVLT) [84] (*n* = 3), the verbal learning and delayed recall scores of the Screen for Cognitive Impairment in Psychiatry (SCIP) [85] (*n* = 1) [23] and the Rey Auditory Verbal Learning Test (RAVLT) [86].

Half of the studies found significant associations with CM, meaning that those with BD and higher CM had worse memory functioning [23, 59, 60, 62].

Random‐effect meta‐analyses showed that only overall CM and physical neglect were negatively associated with verbal memory and learning (*k* = 5; *r* = −0.14, *p* < 0.001; *k* = 4; *r* = −0.18, *p* = 0.006; respectively).

#### 3.4.4 Working Memory

Studies [23, 59, 62, 67, 68, 75] evaluated working memory in BD as measured by the Letter/Number Sequencing (LNS) (*n* = 3) [23, 44, 59, 61], Digit Span Forwards/Backwards (*n* = 2) [67, 68], Arithmetic (*n* = 1) [62] tasks of the WAIS‐III, and the Spatial Working Memory Test of the Cambridge Neuropsychological Test Automated Battery (CANTAB) (*n* = 2) [23, 59].

Most of the studies identified a negative association with CM [23, 59, 62, 67, 68], highlighting that physical or emotional abuse, sexual abuse [23] or physical neglect in particular [67, 68] were associated with a reduction in working memory function. One study did not find such an association [75].

Random‐effect meta‐analyses showed that all CM subtypes (except overall CM and emotional neglect) were negatively associated with working memory (*k* = 4; *r* = −0.12 to −0.16, *p* = 0.042 to < 0.001).

#### 3.4.5 Executive Functions and Verbal Fluency

Studies examined executive functions [23, 43, 59, 60, 62, 67, 68, 69, 71] as a measure of response inhibition using the Wisconsin Card‐Sorting Test (WCST) [87] (*n* = 3) [61, 69, 71], the SCWT [81] (Interference score), and the inhibition control/non‐planning tasks of the Barratt Impulsiveness Scale (BIS‐11) [88], and explored verbal fluency as measured by the Letter Fluency task (*n* = 3) [23, 67, 68], the verbal fluency composite score of the SCIP [85], and the FAS phonemic fluency task of the Controlled Oral Word Association (COWAT) [89].

Overall, studies [61, 69, 71] found an association between CM and impaired executive functions in BD, and dysfunction in verbal fluency [23, 67, 68]. In contrast, four studies [43, 44, 59, 60] did not find any associations between CM and executive functions and/or verbal fluency in BD.

Random‐effect meta‐analyses showed that physical abuse, sexual abuse, and physical neglect were negatively associated with executive functions and verbal fluency (*k* = 6; *r* = −0.07 to −0.13, *p* = 0.037 to < 0.001).

#### 3.4.6 Social Cognition

Studies examined social cognition [43, 44, 60, 73, 74, 75] including *emotion perception* and *theory of mind* (ToM) skills using the Awareness of Social Inference Test (TASIT) [90], the Facial Expressions of Emotion Stimuli test (FEEST) [91] (*n* = 2) [43, 44], the Emotion Perception Test [92] (*n* = 1) [60], the Emotion Recognition Task [93] of the CANTAB (*n* = 1) [65], the DEZICO [94]—ToM index (*n* = 1) [74], the Hinting Task [95] test (*n* = 1) [75] and the MSCEIT [96] (*n* = 1) [62].

Two studies [43, 44] found disturbances in ToM skills but not emotion processing in those with BD and CM exposure. Likewise, one study [60] found no association between CM and facial emotion recognition. While another study [65] found that patients with a positive childhood history of emotional neglect performed worse than those without such a history only in recognising anger. A recent study [74] found poorer ToM to be associated with CM in remitted patients with BD. In contrast, one study [75] found a positive association between CM and ToM [95]. Further, one study [62] found that CM was associated with impaired emotion intelligence.

Random‐effect meta‐analyses showed no significant associations between CM (overall/subtypes) and social cognition of emotion perception and ToM skills.

### 3.5 Heterogeneity, Meta‐Regression Analyses

Meta‐analyses showed zero to low heterogeneity in results for most associations; however, 10 results showed moderate or high heterogeneity (> 50%) and *Q*‐test *p* value < 0.05, of which most results were in social cognition (Table 2).

As the number of included studies was small, no multiple meta‐regressions were computed, and each possible predictor (or moderator) was analysed separately. Associations between CM and cognitive outcomes were largely independent from our pre‐defined variables, with a few exceptions, that is, including six moderating effects with sample size, study quality and sex modifying associations between different CM types and cognitive outcomes (see results of meta‐regressions in SA9 in the Data S1).

### 3.6 Sensitivity Analysis, Publication Bias

To further assess possible causes of heterogeneity and robustness of findings, one‐study‐removed sensitivity analyses were conducted. Removal of single effect sizes did not change the patterns of most results with a few exceptions (see results of each leave‐out sensitivity analysis described in detail and illustrated in SA10 in the Data S1).

Because of the limited number of included studies (*n* < 10) [97], it was not possible to assess the publication bias for any of the associations.

### 3.7 Narrative Synthesis of Moderators and Mediators Reported in the Included Studies

Seven of the included studies [23, 44, 61, 62, 66, 70, 73, 75] investigated effect moderation, and one study [67] investigated effect mediation between CM and cognitive outcomes (see a full description of the main results in SA11 in the Data S1).

## 4 Discussion

This systematic review and meta‐analysis investigated associations between overall and different subtypes of CM, global cognition and IQ, and domains of cognitive functioning in individuals with BD. Across the identified studies, we confirmed overall CM was associated with cognitive functioning in BD. Specifically, overall CM was associated with poorer global cognition and IQ, attention and processing speed, and verbal memory and learning, but not working memory, executive functions, and verbal fluency or social cognition. We also found negative associations between different CM subtypes (except for emotional neglect) and both global cognition/IQ and most cognitive domains. However, associations were overall small magnitude, and findings differed in consistency depending on the CM subtype and cognitive domain considered, suggesting differential and specific effects.

The observed associations between higher CM exposure and poorer cognition/IQ are in line with a substantial literature documenting similar effects across multiple psychiatric conditions [23, 24, 98], as well as wider clinical and neurobiological consequences of CM [18, 19, 99] across these conditions. Long‐term elevation of cortisol levels and hypothalamic pituitary adrenal (HPA) axis dysfunction during childhood, and exaggerated systemic and intracellular inflammatory responses to repeated psychosocial stress in adulthood [27, 100] are potential underlying mechanisms of these negative effects of CM and BD on cognitive functions.

Exposure to overwhelming stress from CM, particularly abuse and neglect, can lead to sustained cortisol elevation that negatively impacts brain development and contributes to cognitive impairments across multiple domains [101, 102, 103]. This chronic cortisol elevation, along with altered neurodevelopment, potentially mediates the relationship between CM and cognitive deficits. Supporting this, CM has been associated with structural brain changes, particularly in the prefrontal cortex [21] and white matter [104], which are regions with prolonged developmental timelines that are essential for cognitive function. The finding that individuals with BD showed sensitivity to the cognitive impact of CM aligns with the diathesis‐stress model [105], indicating that both BD and CM might contribute to worse cognitive performance through potential synergistic effects or interaction between intrinsic vulnerability (such as genetic variants) and external stressors (such as CM), with the effects of CM extending broadly to general cognition. This observation aligns with previous findings showing CM‐related cognitive impairments in both clinical and non‐clinical populations [106, 107].

Although the effect size of the relationship between CM and cognitive functioning was fairly small, low heterogeneity lends support to the robustness of the findings. Nonetheless, a critical future direction will be to determine whether this effect is clinically meaningful. Given the vast evidence that CM increases the likelihood of developing BD [108] and that cognitive deficits are a core component of the disorder [3], it is also possible that a larger effect is being constrained by methodological limitations in the literature. In the present investigation, however, there was no evidence of (consistent) methodological moderator effects. We also need to consider that some of the significant results found in this review may be affected by confounding variables not addressed by most of the included studies (e.g., duration of illness and substance use), and that there could be other, non‐causal explanations, such as poverty could increase the risk of CM exposure and cognitive functioning impairment [109, 110]. Most of the identified studies were cross‐sectional; whether associations between CM and (social) cognitive functioning in BD may in fact be bidirectional should be examined in future prospective studies.

Our findings highlight the significant role of early childhood experiences in cognitive development among individuals with BD, challenging traditional views that cognitive deficits are purely genetic. BD itself impacts neurocognition independently of childhood trauma or family history [3, 111], though further replication is needed to confirm this. Results suggest that the combination of CM and BD may exacerbate cognitive vulnerabilities already present in BD, implicating both genetic and environmental factors in these deficits. The associations with CM were weak, suggesting that cognitive impairments in BD are likely influenced by additional factors—such as medication [112], relapses [113], and social factors like support and isolation [114].

Although small, the effect size associated with attention and processing speed and overall CM was among the largest observed across cognitive domains. Additionally, four out of five CM subscales showed significant associations with working memory. This is in line with prior meta‐analytic evidence on the link between CM and neurocognition in adults with psychosis [24] and suggests that exposure to CM can worsen performance on tasks that require skills using complex information to make decisions. Furthermore, this is notable especially in light of the fact that processing speed and working memory have been considered neurocognitive endophenotypes for BD [115, 116]. The current evidence does not allow for directly observing the mechanisms associated with these relationships. However, this is an important first step for future examinations that may allow a stronger understanding of the distinct domains and neural correlates underlying the observed associations.

Interestingly, the physical types of CM showed the strongest associations with worse cognitive functioning in BD. This is consistent with evidence on specific trauma types effects on cognitive abilities [28], and that associations with cognitive deficits were specific to measures of physical abuse or neglect [117, 118]. In turn, there is evidence that among youth with BD, physical abuse is associated with a worse global family environment, more severe depressive and manic symptoms, a greater likelihood of suicidality and of being diagnosed with post‐traumatic stress disorder, and more alcohol or drug use [119], all factors that may mediate the relationship between CM and cognitive functioning of BD. Taken together, our findings indicate that physical maltreatment represents an important (early) intervention target for BD.

We corroborate previous findings showing mixed results and no strong link between CM and social cognition, particularly in ToM and emotion processing, and that sex moderates this association in BD [26]. Future research should explore CM effects in relation to timing [120] and sensitive periods [120] on neurodevelopment and cognitive vulnerability in BD, preferably employing multimodal approaches (e.g., neuroimaging and clinical assessments [121]) to capture the role of neurobiological factors and psychosocial influences, like cognitive reserve [122] and resilience [123].

Despite the relevance of CM in BD, studies examining its effects on cognitive outcomes are scarce, particularly in BD Type II and first‐episode cases. Although CM prevalence may be higher in BD than in other mental disorders [17], it is less frequently recognised in BD [124]. Nonetheless, the recent increase in publications on CM in BD underscores the growing recognition of its importance. Further research (in larger samples) on the role of physical versus emotional CM on cognitive functiong in BD is crucial to inform interventions and improve outcomes. See also Table 3 for a summary of methodological issues and further recommendations for future studies in this area.

**TABLE 3:** Methodological problems identified in the included studies and recommendations for future research.

Methodological problem	Recommendation
Lack of studies measuring different types and timing of CM exposure	Studies should use the MACE [125] to assess abuse, neglect, domestic violence and bullying, age at onset and duration of CM
Lack of daily‐life cognitive skills assessment	Use virtual reality as the CAVIR [126] test
Lack of studies assessing social cognition	Investigate emotion processing using valanced, naturalistic stimuli – including faces, scenes, and interactions [121]
With cross‐sectional studies is not possible to imply causation	Longitudinal cohort studies with early life recruitment and international collaboration where possible
Statistical analyses for multiple outcomes and low power	Use adequately powered sample sizes Correct for multiple outcomes to avoid type one errors
Studies not considering the effects of other stressful events and trauma in adulthood	Include a measure of adulthood trauma such as the ITQ [127]
Inconsistencies in the screening for comorbidities (suicide, substance abuse)	Screen for psychiatric comorbidities with brief measures as the MINI [128]. Consider including PTSD in analyses [13]
Lack of studies reporting full‐sociodemographic characteristics	Report SES, gender, social support [129]
Lack of studies reporting full‐clinical characteristics	Report number of episodes, BD type, state (remission vs. in episode), IQ
Lack of studies including confounders, and potential moderating/mediating factors in the association between CM and cognitive outcomes in BD	Include covariates such as IQ, SES, mood symptoms/status [25], onset and duration of CM and BD, substance and medication use. Include moderation/mediation analyses involving sex/gender [130], genetic/epigenetic factors, brain structure and function [104], physical activity, personality traits, attachment, social support [129] and resilience domains [123]

Abbreviations: BD = Bipolar disorder; CAVIR = Cognition Assessment in Virtual Reality; CM = Childhood maltreatment; IQ = Intelligence quotient; ITQ = International Trauma Questionnaire; MACE = Maltreatment and Abuse Chronology of Exposure; MINI = Mini‐International Neuropsychiatric Interview; PTSD = Posttraumatic stress disorder; SES = Socioeconomic status.

### 4.1 Clinical Implications

Clinically, although our findings provide preliminary evidence of poorer cognitive functioning in BD patients with a history of CM, they align with a growing body of research suggesting that CM should be routinely considered during assessment, diagnosis, and treatment of BD. Assessing CM and cognition systematically in clinical settings could support early intervention, mitigate cognitive impact and may even contribute to more accurate diagnoses. While some institutions already include CM in standard assessments, broader adoption of this practice across mental health settings would strengthen preventive and supportive care, particularly by addressing CM‐related cognitive impairment early in the illness.

Our findings further suggest that trauma‐informed interventions, coupled with cognitive remediation and skills training, may help to offset the negative impact of CM on cognitive functioning throughout life. Psychosocial interventions addressing affective instability [131], alongside training in attention and cognitive skills, are also recommended to support sustained cognitive resilience in patients exposed to early stress. Additionally, early interventions aimed at children in families with psychiatric disorders could help prevent CM, improving long‐term cognitive development and reducing the risk of depressive symptoms in paediatric BD. This proactive approach could have far‐reaching benefits for cognitive and emotional stability over the lifespan.

### 4.2 Strengths and Limitations

This study builds on the well‐established role of CM in the risk of BD by acknowledging that experiences of CM could also be related to the cognitive impairment of those diagnosed with BD. This study benefitted from the wide range of pooled subjects, which constitutes a geographically diverse sample with low heterogeneity. Although there was some variability in which types of CM were reported, all but two studies used the same standard and validated instrument to assess CM (CTQ) [76]. Other strengths of this study include the rigorous methodology with the systematic search, study selection and data extraction performed by three independent researchers.

Our work also includes some limitations. First, the number of studies available for meta‐analysis was small and the capacity to identify heterogeneity and moderators was substantially limited. However, we followed the Cochrane recommendations [132] and the number of studies included in meta‐analyses was constrained by the limited number of studies that examined CM and cognitive functioning in BD. Because of the limited number of included studies in some meta‐analyses (*n* < 5) [133] or meta‐regressions (*n* < 10) [97] conducted, analyses should be considered exploratory and interpreted with caution. The sample sizes were often small, meaning that analyses may not have been sufficiently powered for detecting small effects. The extent of the effects of these limitations will become evident as more studies examining these questions become available. Second, it was impossible to account for all the possible variations across instruments utilised, although most studies assessed cognitive outcomes with robust tools. In addition, some of our domains did not include exactly the same measures (e.g., global cognition and IQ, executive functions and verbal fluency, or emotion perception and ToM skills); yet, we followed the same procedure as in previous reports [23, 24, 26, 43, 44] to define and operationalise our cognitive domains. Third, while exclusion of grey literature (or unpublished work) ensured less heterogeneity in study quality, this could also cause relevant findings to be missed and bias for publication of ‘positive’ results. Nevertheless, the inclusion of data from unpublished studies could also introduce bias [134]. Finally, CM was retrospectively reported through assessments that can be biased. However, retrospective self‐reports on the presence of CM by patients with BD showed sufficient reliability [135].

## 5 Conclusions

In conclusion, overall CM and its subtypes (except for emotional neglect) are linked to poorer global cognition and IQ and cognitive impairments across several domains in BD, particularly verbal memory and learning, working memory, executive functions, and verbal fluency, but are unrelated to social cognition. While associations are weak, exploring the timing of CM as well as moderators like attachment, mood symptom severity and genetic factors may clarify these relationships. The need for prospective and interventional studies is critical due to addressing the limitations of the current evidence, which mainly comprises cross‐sectional studies with retrospective reporting of CM. Our findings support CM as a key predictor of cognitive functioning in BD, suggesting the potential benefit of trauma‐informed interventions and cognitive remediation strategies. Early interventions for at‐risk children may help improve cognitive outcomes long‐term, underscoring the importance of identifying CM in BD to improve patient care and quality of life.

## Author Contributions

Protocol registration, Term: N.E.F.‐O. Conceptualisation: N.E.F.‐O., K.W.M., E.V. Data collection/curation: N.E.F.‐O., A.P.‐R., R.L.‐E., S.M.‐P. Writing – original draft: N.E.F.‐O., K.W.M. Writing – reviewing and editing, Interpretation of the data: N.E.F.‐O., L.A., S.L.H., K.W.M., E.V. Methodology, Formal analysis, Software, Validation: N.E.F.‐O. Visualisation: N.E.F.‐O., A.P.‐R., R.L.‐E. Investigation: N.E.F.‐O., A.P.‐R., R.L.‐E. Resources, Funding acquisition: K.W.M., E.V. Project administration: N.E.F.‐O. Supervision: N.E.F.‐O., K.W.M., E.V. All authors revised and approved the final version of the submitted manuscript.

## Conflicts of Interest

E.V. has received grants and served as consultant, advisor or CME speaker for the following entities: AB‐Biotics, AbbVie, Adamed, Angelini, BeckleyPsych, Biogen, Biohaven, Boehringer‐Ingelheim, Celon Pharma, Compass, Dainippon Sumitomo Pharma, Ethypharm, Ferrer, Gedeon Richter, GH Research, Glaxo‐Smith Kline, HMNC, Idorsia, Janssen, Lundbeck, Luye Pharma, Medincell, Merck, Newron, Novartis, Orion Corporation, Organon, Otsuka, Roche, Rovi, Sage, Sanofi‐Aventis, Sunovion, Takeda, Teva, and Viatris, outside of the submitted work. L.A. has received payments for non‐promotional seminars from Alianza Otsuka‐Lundbeck. K.W.M. has received honoraria from Gedeon Richter, Lundbeck and Angelini in the past three years. The other authors report no financial relationships with commercial interests.

## Supporting information


**Data S1.** Supporting Information.

## Data Availability

N.E.F.‐O. has full access to all data in the study and takes responsibility for the integrity of the data and the accuracy of the data analyses. The data that support the findings of this study are available upon reasonable request.
