# Supplementary material for: Childhood Maltreatment and Cognitive Functioning in Bipolar Disorder: A Systematic Review and Meta‐Analysis
Source: Acta Psychiatr Scand. 2025 May 6;153(5):315–38. doi: 10.1111/acps.13813 (PMC13050602; doi:10.1111/acps.13813)
Supplement: Supplementary file 1 — Data S1. Supporting Information. [file ACPS-153-315-s001.docx]

**SUPPLEMENT**

**Childhood maltreatment and cognitive functioning in bipolar disorder: A systematic review and meta-analysis**

[**SA1. Glossary of terms** 1](#_Toc182340228)

[**SA2. Literature search** 4](#_Toc182340229)

[**SA3. Definition of exposure and outcome variables** 11](#_Toc182340230)

[**SA4. Extracted variables** 13](#_Toc182340231)

[**SA5. Full list of included studies** 14](#_Toc182340232)

[**SA6. Excluded studies with reasons** 16](#_Toc182340233)

[**SA7. Forest plots investigating associations between CM and cognitive outcomes** 20](#_Toc182340234)

[**SA8. Qualitative synthesis of cognitive domains and studies not providing sufficient data for meta-analysis** 32](#_Toc182340235)

[**SA9. Meta-regression analyses** 33](#_Toc182340236)

[**SA10. One-study-removed sensitivity analyses** 34](#_Toc182340237)

[**SA11. Narrative synthesis of moderators and mediators reported in the included studies** 37](#_Toc182340238)

[**ST1. PRISMA 2020 statement and checklist** 39](#_Toc182340239)

[**ST2. PRISMA 2020 for abstracts checklist** 44](#_Toc182340240)

[**ST3. MOOSE guideline and checklist** 46](#_Toc182340241)

[**ST4. Manuals codes for bipolar disorder diagnoses included** 50](#_Toc182340242)

[**ST5. Description and measurement of cognitive functioning** 51](#_Toc182340243)

[**ST6. Quality assessment of included studies** 62](#_Toc182340244)

[**Newcastle-Ottawa scale for non-randomised studies** 63](#_Toc182340245)

[**Quality assessment score** 65](#_Toc182340246)

[**Results of the study quality assessment of the included studies** 66](#_Toc182340247)

[**REFERENCES** 67](#_Toc182340248)

# **SA1. Glossary of terms**

ACE-R: Addenbrooke's Cognitive Examination Revised

ACT: Turkish version of the Auditory Consonant Trigrams Test

ADHD: Attention deficit hyperactivity disorder

AGN: CANTAB’s Affective Go/No-Go Task

AMT: Autobiographical Memory Test

BAC-A: Brief Assessment of Cognition in Affective Disorders

BD: Bipolar Disorder

BIS-11: Barrat Impulsiveness Scale

CANTAB: Cambridge Neuropsychological Test Automated Battery

CECA-Q: Childhood Experiences of Care and Abuse Questionnaire

CIs: Confidence Intervals

CM: Childhood Maltreatment

CMA: Comprehensive Meta-Analysis

COWAT: Controlled Oral Word Association Test

CPT-II: Continuous Performance Test-II

CTQ: Childhood Trauma Questionnaire

CTQ-SF: Childhood Trauma Questionnaire-Short Form

CVLT: California Verbal Learning Test

DEZIKO: Dokuz Eylul Theory of Mind Index

DIGS: Diagnostic Interview for Genetic Studies

DSCT: Digital Symbol Coding Test

DSM: Diagnostic and Statistical Manual of Mental Disorders

EA: Emotional Abuse

EN: Emotional Neglect

EPT: Emotion Perception Test

EQUATOR: Quality and Transparency of Health Research

ERT: CANTAB’s Emotion Recognition Task

FAS: Word Fluency of Controlled Oral Word Association Test

FEEST: Facial Expressions of Emotion Stimuli and Tests

HPA: Hypothalamic-pituitary-adrenal axis

ICD: International Statistical Classification of Diseases and Related Health Problems

IED: CANTAB’s, Intra-/extra-dimensional test

IQ: Intellectual Quotient

IRI: Interpersonal Reactivity Index

KAS: COWAT’s K-A-S phonemic fluency task

K-BIT: Kaufman Brief Intelligence Test

KSADS: Kiddie Schedule for Affective Disorders and Schizophrenia

KSADS-PL: Kiddie Schedule for Affective Disorders and Schizophrenia Present and Lifetime Version

LEC: Life Events Checklist

LNS: Letter Number Sequencing

MACE: Maltreatment and Abuse Chronology of Exposure Scale

MCQ-30: Metacognition Questionaire-30

MINI: Mini International Neuropsychiatric Interview

MSCEIT 2.0: Mayer-Salovey-Caruso Emotional Intelligence Test Version 2.0

NA: Not available

NART: National Adult Reading Test

NOS: Newcastle Ottawa Scale

NS: Non-Significant

OTS: CANTAB’s One Touch Stockings of Cambridge test

OXTR: Oxytocin Receptor Gene

PA: Physical Abuse

PAL: CANTAB’s Paired Associate Learning test

PGNG: Parametric Go/No-Go

PN: Physical neglect

PRISMA: Preferred Reporting Items for Systematic Reviews and Meta-Analyses

PRM: CANTAB’s Pattern Recognition Memory test

RAVLT: Rey-Auditory Verbal Learning Test

RBANS: Repeatable Battery for the Assessment of Neuropsychological Status

ROCF: Rey-Osterrieth Complex Figure Test

RT: Reaction Time

RVIP: CANTAB’s Rapid Visual Information Processing test

RVP: Rapid Visual Processing Task

SA: Sexual Abuse

SCAN: Schedules for Clinical Assessment in Neuropsychiatry

SCID: Structured Clinical Interview for DSM

SCID-5: The Structured Clinical Interview for DSM-5

SCID-I: The Structured Clinical Interview for DSM-IV Axis I Disorders

SCIP: Screen for Cognitive Impairment in Psychiatry

SCWT: Stroop Colour-Word Test

SD: Standard Deviation

SES: Socioeconomic Status

SRM: CANTAB’s Spatial Recognition Memory Test

SWM: CANTAB’s Spatial Working Memory Test

TASIT: Awareness of Social Interference Test

TEC: Traumatic Experience Checklist

TFR: Test of Facial Recognition

THQ: Trauma History Questionnaire

TMT: Trail Making Test

ToM: Theory of Mind

VCT: WMS-R’s Visual Copying Test

WAIS: Wechsler Adult Intelligence Scale

WASI: Wechsler Abbreviated Scale of Intelligence

WCST: Wisconsin Card Sort Test

WM: Wechsler Adult Intelligence Scale-III, Working memory index

WMS: Wechsler Memory Scale

# **SA2. Literature search**

**Date of the searches: December 1^st^, 2023, updated on June 24^th^, 2024.**

**1) PsycINFO**

**129 articles retrieved + 120 articles retrieved**

#1 Title = cognitive OR cognition OR neurocognitive OR neurocognition OR neuropsychological OR memory OR attention OR "executive functioning" OR "executive functions" OR "fluency" OR "decision making" OR "problem solving" OR reasoning OR visuoconstructive OR visuospatial OR "processing speed" OR "inhibitory control" OR intelligence OR "perceptual speed" OR "processing speed"OR "verbal learning" OR "social cognition" OR "theory of mind" OR "face perception" OR "face discrimination" OR "face recognition" OR "emotion recognition" OR "emotion processing" OR "attributional style" OR "social perception" OR "social knowledge" OR "cold cognition" OR "hot cognition" OR IQ OR ToM OR "emotional processing" OR "emotional intelligence"

#2 Abstract = cognitive OR cognition OR neurocognitive OR neurocognition OR neuropsychological OR memory OR attention OR "executive functioning" OR "executive functions" OR "fluency" OR "decision making" OR "problem solving" OR reasoning OR visuoconstructive OR visuospatial OR "processing speed" OR "inhibitory control" OR intelligence OR "perceptual speed" OR "processing speed" OR "verbal learning" OR "social cognition" OR "theory of mind" OR "face perception" OR "face discrimination" OR "face recognition" OR "emotion recognition" OR "emotion processing" OR "attributional style" OR "social perception" OR "social knowledge" OR "cold cognition" OR "hot cognition" OR IQ OR ToM OR "emotional processing" OR "emotional intelligence"

#3 #1 OR #2

#4 Title ="child abuse" OR "childhood abuse" OR "child neglect" OR "childhood neglect" OR "physical abuse" OR "physical neglect" OR "sexual abuse" OR "emotional abuse" OR "emotional neglect" OR "child maltreatment" OR "childhood maltreatment" OR "childhood trauma" OR "psychological trauma" OR "psychological maltreatment" OR "childhood adversities" OR "early life adversity" OR "early life stress" OR "domestic violence" OR "parental discord" OR bullying OR "interpersonal violence" OR "interpersonal trauma" OR CTQ OR "childhood trauma questionnaire" OR CECA

#5 Abstract ="child abuse" OR "childhood abuse" OR "child neglect" OR "childhood neglect" OR "physical abuse" OR "physical neglect" OR "sexual abuse" OR "emotional abuse" OR "emotional neglect" OR "child maltreatment" OR "childhood maltreatment" OR "childhood trauma" OR "psychological trauma" OR "psychological maltreatment" OR "childhood adversities" OR "early life adversity" OR "early life stress" OR "domestic violence" OR "parental discord" OR bullying OR "interpersonal violence" OR "interpersonal trauma" OR CTQ OR "childhood trauma questionnaire" OR CECA

#6 #4 OR #5

#7 Title ="bipolar disorder" OR mania OR hypomania OR "mood disorder" OR "affective disorder" OR "bipolar depression"

#8 Abstract ="bipolar disorder" OR mania OR hypomania OR "mood disorder" OR "affective disorder" OR "bipolar depression"

#9 #6 OR #7

#10 #3 AND #6 AND #9

#11 #10 AND Articles

**2) Embase**

**129 articles retrieved + 12 articles retrieved**

#1 cognitive:ti,ab OR cognition:ti,ab OR neurocognitive:ti,ab OR neurocognition:ti,ab OR neuropsychological:ti,ab OR 'executive functioning':ti,ab OR 'executive functions':ti,ab OR fluency:ti,ab OR 'decision making':ti,ab OR 'problem solving':ti,ab OR reasoning:ti,ab OR visuoconstructive:ti,ab OR visuospatial:ti,ab OR 'inhibitory control':ti,ab OR intelligence:ti,ab OR 'perceptual speed':ti,ab OR 'processing speed':ti,ab OR 'verbal learning':ti,ab OR 'social cognition':ti,ab OR 'theory of mind':ti,ab OR 'face perception':ti,ab OR 'face discrimination':ti,ab OR 'face recognition':ti,ab OR 'emotion recognition':ti,ab OR 'emotion processing':ti,ab OR 'attributional style':ti,ab OR 'social perception':ti,ab OR 'social knowledge':ti,ab OR 'cold cognition':ti,ab OR 'hot cognition':ti,ab OR iq:ti,ab OR tom:ti,ab OR 'emotional processing':ti,ab OR 'emotional intelligence':ti,ab

#2 'memory'/exp

#3 #1 OR #2

#4 'child abuse'/exp

#5 'childhood abuse':ti,ab OR 'child neglect':ti,ab OR 'childhood neglect':ti,ab OR 'physical abuse':ti,ab OR 'physical neglect':ti,ab OR 'sexual abuse':ti,ab OR 'emotional abuse':ti,ab OR 'emotional neglect':ti,ab OR 'child maltreatment':ti,ab OR 'childhood maltreatment':ti,ab OR 'childhood trauma':ti,ab OR 'psychological trauma':ti,ab OR 'psychological maltreatment':ti,ab OR 'childhood adversities':ti,ab OR 'early life adversity':ti,ab OR 'early life stress':ti,ab OR 'domestic violence':ti,ab OR 'parental discord':ti,ab OR bullying:ti,ab OR 'interpersonal violence':ti,ab OR 'interpersonal trauma':ti,ab OR ctq:ti,ab OR 'childhood trauma questionnaire':ti,ab OR ceca:ti,ab

#6 #4 OR #5

#7 "bipolar disorder":ti,ab OR mania:ti,ab OR hypomania:ti,ab OR "mood disorder":ti,ab OR "affective disorder":ti,ab OR "bipolar depression":ti,ab

#8 #3 AND #6 AND #7

#9 #8 AND ('article'/it OR 'article in press'/it OR 'preprint'/it OR 'review'/it OR 'short survey'/it)

**3) PubMed**

**126 articles retrieved + 13 articles retrieved**

#1 cognitive[tiab] OR cognition[tiab] OR neurocognitive[tiab] OR neurocognition[tiab] OR neuropsychological[tiab] OR "Memory"[MeSH] OR "Attention"[MeSH] OR "executive functioning"[tiab] OR "executive functions"[tiab] OR "fluency"[tiab] OR "decision making"[tiab] OR "problem solving"[tiab] OR reasoning[tiab] OR visuoconstructive[tiab] OR visuospatial[tiab] OR "processing speed"[tiab] OR "inhibitory control"[tiab] OR intelligence[tiab] OR "perceptual speed"[tiab] OR "processing speed"[tiab] OR "verbal learning"[tiab] OR "social cognition"[tiab] OR "theory of mind"[tiab] OR "face perception"[tiab] OR "face discrimination"[tiab] OR "face recognition"[tiab] OR "emotion recognition"[tiab] OR "emotion processing"[tiab] OR "attributional style"[tiab] OR "social perception"[tiab] OR "social knowledge"[tiab] OR "cold cognition" [tiab] OR "hot cognition"[tiab] OR IQ [tiab] OR ToM [tiab] OR "emotional processing" [tiab] OR "emotional intelligence" [tiab]

#2 "child abuse"[MeSH] OR "childhood abuse"[tiab] OR "child neglect"[tiab] OR "childhood neglect"[tiab] OR "physical abuse"[tiab] OR "physical neglect"[tiab] OR "sexual abuse"[tiab] OR "emotional abuse"[tiab] OR "emotional neglect"[tiab] OR "child maltreatment"[tiab] OR "childhood maltreatment"[tiab] OR "childhood trauma"[tiab] OR "psychological trauma"[tiab] OR "psychological maltreatment"[tiab] OR "childhood adversities"[tiab] OR "early life adversity"[tiab] OR "early life stress"[tiab] OR "domestic violence"[tiab] OR "parental discord"[tiab] OR bullying[tiab] OR "interpersonal violence"[tiab] OR "interpersonal trauma"[tiab] OR CTQ[tiab] OR "childhood trauma questionnaire"[tiab] OR CECA[tiab]

#3 "bipolar disorder"[tiab] OR mania[tiab] OR hypomania[tiab] OR "mood disorder"[tiab] OR "affective disorder"[tiab] OR "bipolar depression" [tiab]

#4 #1 AND #2 AND #3

**4) Web of Science (Core collection)**

**120 articles retrieved + 6 articles retrieved**

#1 TI=(cognitive OR cognition OR neurocognitive OR neurocognition OR neuropsychological OR "executive functioning" OR "executive functions" OR fluency OR "decision making" OR "problem solving" OR reasoning OR visuoconstructive OR visuospatial OR "processing speed" OR "inhibitory control" OR intelligence OR "perceptual speed" OR "processing speed" OR "verbal learning" OR "social cognition" OR "theory of mind" OR "face perception" OR "face discrimination" OR "face recognition" OR "emotion recognition" OR "emotion processing" OR "attributional style" OR "social perception" OR "social knowledge" OR "cold cognition" OR "hot cognition" OR IQ OR ToM OR "emotional processing" OR "emotional intelligence" OR memory )

#2 AB=(cognitive OR cognition OR neurocognitive OR neurocognition OR neuropsychological OR "executive functioning" OR "executive functions" OR fluency OR "decision making" OR "problem solving" OR reasoning OR visuoconstructive OR visuospatial OR "processing speed" OR "inhibitory control" OR intelligence OR "perceptual speed" OR "processing speed" OR "verbal learning" OR "social cognition" OR "theory of mind" OR "face perception" OR "face discrimination" OR "face recognition" OR "emotion recognition" OR "emotion processing" OR "attributional style" OR "social perception" OR "social knowledge" OR "cold cognition" OR "hot cognition" OR IQ OR ToM OR "emotional processing" OR "emotional intelligence" OR memory )

#3 #1 OR #2

#4 TI=("childhood abuse" OR "child neglect" OR "childhood neglect" OR "physical abuse" OR "physical neglect" OR "sexual abuse" OR "emotional abuse" OR "emotional neglect" OR "child maltreatment" OR "childhood maltreatment" OR "childhood trauma" OR "psychological trauma" OR "psychological maltreatment" OR "childhood adversities" OR "early life adversity" OR "early life stress" OR "domestic violence" OR "parental discord" OR bullying OR "interpersonal violence" OR "interpersonal trauma" OR CTQ OR "childhood trauma questionnaire" OR CECA)

#5 AB=("childhood abuse" OR "child neglect" OR "childhood neglect" OR "physical abuse" OR "physical neglect" OR "sexual abuse" OR "emotional abuse" OR "emotional neglect" OR "child maltreatment" OR "childhood maltreatment" OR "childhood trauma" OR "psychological trauma" OR "psychological maltreatment" OR "childhood adversities" OR "early life adversity" OR "early life stress" OR "domestic violence" OR "parental discord" OR bullying OR "interpersonal violence" OR "interpersonal trauma" OR CTQ OR "childhood trauma questionnaire" OR CECA)

#6 #4 OR #5

#7 TI=("bipolar disorder" OR mania OR hypomania OR "mood disorder" OR "affective disorder" OR "bipolar depression" )

#8 AB=("bipolar disorder" OR mania OR hypomania OR "mood disorder" OR "affective disorder" OR "bipolar depression" )

#9 #7 OR #8

#10 #3 AND #6 AND #9

#11 #10 AND Article OR “Review Article OR Editorial Material

**5) Cochrane**

**90 articles retrieved + 6 articles retrieved**

#1 cognitive OR cognition OR neurocognitive OR neurocognition OR neuropsychological OR "executive functioning" OR "executive functions" OR fluency OR "decision making" OR "problem solving" OR reasoning OR visuoconstructive OR visuospatial OR "processing speed" OR "inhibitory control" OR intelligence OR "perceptual speed" OR "processing speed" OR "verbal learning" OR "social cognition" OR "theory of mind" OR "face perception" OR "face discrimination" OR "face recognition" OR "emotion recognition" OR "emotion processing" OR "attributional style" OR "social perception" OR "social knowledge" OR "cold cognition" OR "hot cognition" OR IQ OR ToM OR "emotional processing" OR "emotional intelligence"

#2 "Memory"[MeSH]

#3 “Attention"[MeSH]

#4 #1 OR #2 OR #3

#5 "child abuse"[MeSH]

#6 "childhood abuse" OR "child neglect" OR "childhood neglect" OR "physical abuse" OR "physical neglect" OR "sexual abuse" OR "emotional abuse" OR "emotional neglect" OR "child maltreatment" OR "childhood maltreatment" OR "childhood trauma" OR "psychological trauma" OR "psychological maltreatment" OR "childhood adversities" OR "early life adversity" OR "early life stress" OR "domestic violence" OR "parental discord" OR bullying OR "interpersonal violence" OR "interpersonal trauma" OR CTQ OR "childhood trauma questionnaire" OR CECA

#7 #5 OR #6

#8 "bipolar disorder" OR mania OR hypomania OR "mood disorder" OR "affective disorder" OR "bipolar depression"

#9 #4 AND #7 AND #8

**6) PILOTS**

**18 articles retrieved + 1 articles retrieved**

#1 cognitive OR cognition OR neurocognitive OR neurocognition OR neuropsychological OR "executive functioning" OR "executive functions" OR fluency OR "decision making" OR "problem solving" OR reasoning OR visuoconstructive OR visuospatial OR "processing speed" OR "inhibitory control" OR intelligence OR "perceptual speed" OR "processing speed" OR "verbal learning" OR "social cognition" OR "theory of mind" OR "face perception" OR "face discrimination" OR "face recognition" OR "emotion recognition" OR "emotion processing" OR "attributional style" OR "social perception" OR "social knowledge" OR "cold cognition" OR "hot cognition" OR IQ OR ToM OR "emotional processing" OR "emotional intelligence" OR memory

#2 “childhood abuse" OR "child neglect" OR "childhood neglect" OR "physical abuse" OR "physical neglect" OR "sexual abuse" OR "emotional abuse" OR "emotional neglect" OR "child maltreatment" OR "childhood maltreatment" OR "childhood trauma" OR "psychological trauma" OR "psychological maltreatment" OR "childhood adversities" OR "early life adversity" OR "early life stress" OR "domestic violence" OR "parental discord" OR bullying OR "interpersonal violence" OR "interpersonal trauma" OR CTQ OR "childhood trauma questionnaire" OR CECA

#3 "bipolar disorder" OR mania OR hypomania OR "mood disorder" OR "affective disorder" OR "bipolar depression"

#4 #1 AND #2 AND #3

# **SA3. Definition of exposure and outcome variables**

**Exposure**

Childhood maltreatment^1^ was defined and operationalised as: (a) Overall CM (or total CM) - cumulative scores of abuse and neglect; (b) Physical abuse defined as acts of violence causing physical harm or injury, including physical punishment; (c) Sexual abuse defined as sexual acts including intercourse or touching toward a child; (d) Emotional abuse defined as verbal or behavioural assaults toward a child that might result in trauma including any humiliating name-calling by an adult or caregiver; (e) Physical neglect defined as caregivers’ failure to provide basic physical needs for the child including shelter, food, clothing or health-care; and (f) Emotional neglect defined as caregivers’ failure to meet the child’s fundamental emotional and psychological needs, including love, care, support, and belonging (see CM assessment and measures in the included studies in Table 1).

**Outcomes**

Cognitive functioning outcomes were defined based on outcomes examined in the included studies, and on categorisations used in previous systematic reviews and meta-analysis in the field^2–4^ as:

**(1) Global cognition and IQ:** this cognitive domain includes a general cognitive ability encompassing various cognitive domains such as memory, attention, executive functions, language, and visuospatial skills^1^. Global cognition is measured by multiple cognitive tests, such as WAIS or WASI, RBANS or CANTAB providing a global composite score. The IQ is also typically presented as a composite score that summarises performance across diverse cognitive tasks^5^.

**(2) Attention and processing speed:** this cognitive domain encompasses various subcomponents of attention, including sustained attention, the ability to maintain focus over prolonged periods; selective attention, the ability to focus on relevant stimuli while filtering out distractions; and divided attention, the capacity to process multiple sources of information simultaneously, and **processing speed,** referring to the capacity to identify, integrate, and respond quickly to visual or verbal information^6^. This domain is typically assessed with the TMT-A, a quick assessment often used to measure both cognitive processing speed and visual attention.

**(3) Verbal memory and learning:** this cognitive domain integrates verbal memory, typically assessed using tasks that require recall or recognition of verbal stimuli^7^ and learning, which is the process through which individuals acquire new information or skills through experience, practice, or instruction^8^.

**(4) Working memory:** this domain refers to the cognitive system responsible for the temporary storage and manipulation of information necessary for complex cognitive tasks such as reasoning, comprehension, and learning. It encompasses the ability to hold and process information over short periods, integrating new information with existing knowledge to guide decision-making and behaviour^9,10^, typically assessed with the LNS subtest of the WAIS.

**(5) Executive functions and verbal fluency:** this cognitive domain involves a set of higher-order cognitive processes that enable goal-directed behaviour, problem-solving, and adaptive responses to novel or complex situations, including cognitive abilities such as planning, inhibitory control, cognitive flexibility, and task switching^7,11,12^. Verbal fluency is the capacity to generate words rapidly and efficiently within specific constraints, reflecting lexical retrieval, language production, and cognitive flexibility, and is also considered an executive function skill^8^, frequently evaluated through test of phonological and semantic fluency (e.g., COWAT).

**(6) Social cognition:** this cognitive domain include processes to perceive, infer, and decode social information and abilities to "make sense of others' behavior"^13,14^, including theory of mind (or mentalising) as the ability to reason about mental states and understand intentions, dispositions, emotions, and beliefs of both oneself and others; and emotion processing including recognition and managing emotions^2^.

# **SA4. Extracted variables**

Descriptive variables extracted included first author and publication year, country/region, sample size, mean age (with standard deviation), sex (percentage of males in the sample), gender, education level (in years; % high-university), socioeconomic status (SES) (% high level), premorbid IQ, study design, type of diagnosis in the sample (BD type I and/or II), type and instrument for diagnosis (and criteria), % of euthymic or remitted patients, mean age at BD onset, duration of the illness (in years), number of (manic and depressive) episodes, psychopharmacological treatment or medication use (% Lithium), psychological or any other psychosocial treatment or intervention (if available), CM instrument used and type of CM exposure reported (overall CM and/or subtypes), timing and duration of CM exposure, instrument/measure to assess cognitive functioning, cognitive outcomes, valence of the outcomes, results on the association between CM and cognitive functioning (including *p* value, effect size, and descriptive summary), confounders, moderators, and mediators investigated in the included studies (if reported).

# **SA5. Full list of included studies**

1. Aas M, Steen NE, Agartz I, et al. Is cognitive impairment following early life stress in severe mental disorders based on specific or general cognitive functioning? Psychiatry Research. 2012;198(3):495-500. doi:10.1016/j.psychres.2011.12.045
2. Arat-Çelik HE, Tuna G, Kök-Kendirlioğlu B, et al. Childhood trauma and neurocognitive functions in individuals with bipolar disorder and unaffected siblings of individuals with bipolar disorder. Neuroscience Applied. 2023;2:101138. doi:10.1016/j.nsa.2023.101138
3. Bücker J, Kozicky J, Torres IJ, et al. The impact of childhood trauma on cognitive functioning in patients recently recovered from a first manic episode: Data from the Systematic Treatment Optimization Program for Early Mania (STOP-EM). Journal of Affective Disorders. 2013;148(2-3):424-430. doi:10.1016/j.jad.2012.11.022
4. Ehrlich TJ, Kim H, Ryan KA, et al. Childhood trauma relates to worse memory functioning in bipolar disorder. Journal of Affective Disorders. 2023;333:377-383. doi:10.1016/j.jad.2023.04.056
5. Hsieh YT, Wu R, Tseng HH, et al. Childhood neglect is associated with corticostriatal circuit dysfunction in bipolar disorder adults. Psychiatry Research. 2021;295:113550. doi:10.1016/j.psychres.2020.113550
6. Jiménez E, Solé B, Arias B, et al. Impact of childhood trauma on cognitive profile in bipolar disorder. Bipolar Disorders. 2017;19(5):363-374. doi:10.1111/bdi.12514
7. *Larsen EM, Ospina LH, Cuesta-Diaz A, et al. Effects of childhood trauma on adult moral decision-making: Clinical correlates and insights from bipolar disorder. Journal of Affective Disorders. 2019;244:180-186. doi:10.1016/j.jad.2018.10.002
8. Lima IMM, Malloy-Diniz LF, de Miranda DM, Da Silva AG, Neves FS, Johnson SL. Integrative Understanding of Familial Impulsivity, Early Adversity and Suicide Risk. Front Psychol. 2017;8. doi:10.3389/fpsyg.2017.02240
9. Martins DS, Hasse-Sousa M, Petry-Perin C, et al. Perceived childhood adversities: Impact of childhood trauma to estimated intellectual functioning of individuals with bipolar disorder. Psychiatry Research. 2019;274:345-351. doi:10.1016/j.psychres.2019.02.046
10. Miskowiak KW, Hansen KB, Mariegaard J, Kessing LV. Association between childhood trauma, cognition, and psychosocial function in a large sample of partially or fully remitted patients with bipolar disorder and healthy participants. Int J Bipolar Disord. 2023;11(1):31. doi:10.1186/s40345-023-00311-w
11. Morán-Kneer J, Ríos U, Costa-Cordella S, et al. Childhood trauma and social cognition in participants with bipolar disorder: The moderating role of attachment. Journal of Affective Disorders Reports. 2022;9:100359. doi:10.1016/j.jadr.2022.100359
12. *Mowlds W, Shannon C, McCusker CG, et al. Autobiographical memory specificity, depression, and trauma in bipolar disorder. British J Clinic Psychol. 2010;49(2):217-233. doi:10.1348/014466509X454868
13. *Oymak Yenilmez D, Atagün Mİ, Keleş Altun İ, et al. Relationship between Childhood Adversities, Emotion Dysregulation and Cognitive Processes in Bipolar Disorder and Recurrent Depressive Disorder. Turk Psikiyatri Derg. 2021;32(1):8-16. doi:10.5080/u23415
14. Quidé Y, Cohen‐Woods S, O’Reilly N, Carr VJ, Elzinga BM, Green MJ. Schizotypal personality traits and social cognition are associated with childhood trauma exposure. British J Clinic Psychol. 2018;57(4):397-419. doi:10.1111/bjc.12187
15. Ríos U, Morán J, Hermosilla J, et al. The interaction of the oxytocin receptor gene and child abuse subtypes on social cognition in euthymic patients with bipolar disorder type I. Front Psychiatry. 2023;14:1151397. doi:10.3389/fpsyt.2023.1151397
16. Ríos U, Moya PR, Urrejola Ó, et al. El maltrato infantil y su rol en el curso clínico de pacientes con trastorno bipolar. Rev méd Chile. 2020;148(2):204-210. doi:10.4067/s0034-98872020000200204
17. Russo M, Mahon K, Shanahan M, et al. The association between childhood trauma and facial emotion recognition in adults with bipolar disorder. Psychiatry Research. 2015;229(3):771-776. doi:10.1016/j.psychres.2015.08.004
18. Takım U, Sarı S, Gokcay H. “The Relationship Between Childhood Traumas and Social Cognition Through Theory of Mind and Alexithymia in Bipolar Disorder.” Psychol Rep. Published online August 4, 2024:332941241269549. doi:10.1177/00332941241269549
19. Vaughn-Coaxum RA, Merranko J, Birmaher B, et al. Longitudinal course of depressive symptom severity among youths with bipolar disorders: Moderating influences of sustained attention and history of child maltreatment. Journal of Affective Disorders. 2021;282:261-271. doi:10.1016/j.jad.2020.12.078
20. Vreeker A, Abramovic L, Boks MPM, et al. The relationship between brain volumes and intelligence in bipolar disorder. Journal of Affective Disorders. 2017;223:59-64.doi:10.1016/j.jad.2017.07.009

****Note:*** only in qualitative synthesis (systematic review).

# **SA6. Excluded studies with reasons**

| Aas et al. 2013 | Wrong population |
| --- | --- |
| Aas et al. 2019 | Wrong article type |
| Aas et al. 2021 | Wrong study design |
| Azevedo et al. 2024 | Wrong article type |
| Baryshnikov et al. 2018 | Wrong article type |
| Begemann et al. 2023 | Wrong outcome |
| Benedetti et al. 2015 | Wrong outcome |
| Berthelot et al. 2015 | Wrong population |
| Bratlien et al. 2014 | Wrong population |
| Bryan et al. 2014 | Wrong population |
| Bureau et al. 2023 | Wrong population |
| Chamorro et al. 2012 | Wrong population |
| Chan et al. 2019 | Wrong study design |
| ChiCTR2100044665 | Wrong article type |
| ChiCTR2100045265 | Wrong article type |
| Congio et al. 2022 | Association not reported |
| Corcoran et al. 2020 | Wrong exposure |
| CTR 2007-006561-32-DE | Wrong article type |
| Cui et al. 2016 | Wrong population |
| Czepielewski et al. 2022 | Wrong Design |
| Daruy-Filho et al. 2013 | Wrong outcome |
| de Freitas et al. 2024 | Association not reported |
| del Re et al. 2023 | Association not reported |
| Deng and He 2023 | Wrong outcome |
| Dopheide, 2006 | Wrong population |
| Drachman et al. 2022 | Wrong outcome |
| DRKS00010820 | Wrong article type |
| Fijtman et al. 2020 | Association not reported |
| Flechsenhar et al. 2022 | Wrong population |
| Grassi-Oliveira et al. 2008 | Wrong population |
| Green et al. 2014 | Wrong article type |
| Guillaume et al. 2013 | Wrong population |
| Hammersley et al. 2003 | Wrong outcome |
| Hanford et al. 2019 | Wrong population |
| Hsueh et al. 2024 | Association not reported |
| IRCT20171223038002N1 | Wrong article type |
| Janiri et al. 2017 | Wrong outcome |
| Jørgensen et al. 2023 | Overlapping |
| Khosravani et al. 2021 | Wrong outcome |
| Kienhorst et al. 1992 | Wrong population |
| Kim et al. 2019 | Association not reported |
| Kim et al. 2023 | Wrong population |
| Lebovitz et al. 2021 | Association not reported |
| Li et al. 2014 | Wrong outcome |
| Ludwig et al. 2022 | Wrong population |
| Lyall et al. 2018 | Wrong population |
| Maes et al. 2018 | Wrong design |
| Markt et al. 2022 | Association not reported |
| Marshall et al. 2016 | Overlapping |
| Marwaha et al. 2020 | Wrong outcome |
| McCarthy et al. 2020 | Wrong population |
| Moreno-Alcázar et al. 2017 | Wrong article type |
| Morgan et al. 2016 | Wrong population |
| Moser et al. 2018 | Wrong outcome |
| NCT03915613 | Wrong article type |
| NCT03664713 | Wrong article type |
| NL9582 | Wrong article type |
| Oberste et al. 2018 | Wrong population |
| Olgiati et al. 2022 | Wrong population |
| Palagini et al. 2021 | Wrong outcome |
| Palmier-Claus et al. 2016 | Wrong population |
| Parker et al. 2013 | Wrong outcome |
| Perich et al. 2014 | Wrong outcome |
| Poletti et al. 2014 | Wrong outcome |
| Poletti et al. 2017 | Association not reported |
| Post, 2014 | Wrong article type |
| Quidé et al. 2017 | Wrong population |
| Richard-Lepourie et al. 2019 | Wrong outcome |
| Rodrigues de Aguiar et al. 2023 | Wrong population |
| Russo et al. 2014 | Wrong study design |
| Savitz et al. 2007 | Wrong population |
| Savitz et al. 2008 | Association not reported |
| Savitz et al. 2009 | Wrong study design |
| Schwarz et al. 2022 | Wrong article type |
| Scott et al. 2010 | Wrong outcome |
| Serafini et al. 2016 | Wrong outcome |
| Simon et al. 2021 | Wrong article type |
| Sinclair et al. 2007 | Wrong population |
| Strawbridge et al. 2016 | Wrong article type |
| Stumbo et al. 2015 | Wrong outcome |
| Szmulewicz et al. 2020 | Wrong study design |
| Tonini et al. 2022 | Wrong population |
| Toyoshima et al. 2020 | Wrong population |
| Toyoshima et al. 2022 | Wrong population |
| Vaí et al. 2020 | Association not reported |
| van Bergen et al. 2019 | Wrong outcome |
| van der Markt et al. 2022 | Wrong study design |
| Vares et al. 2015 | Wrong population |
| Wang et al. 2021 | Wrong population |
| Yang et al. 2024 | Association not reported |
| Yin et al. 2020 | Wrong population |
| Yin et l. 2021 | Wrong population |
| Zavaschi et al. 2006 | Wrong outcome |
| Zelazny et al. 2019 | Wrong population |
| Zenses et al. 2014 | Wrong article type |
| Zhang et al. 2022 | Wrong outcome |

# **SA7. Forest plots investigating associations between CM and cognitive outcomes**

**Overall CM**

***Note:*** In Ehrlich et al. the SCWT - interference score was used as a measure of executive function.

**Emotional abuse**

***Note:***  In Jiménez et al. the SCWT - interference score was used as a measure of executive function.

**Physical abuse**

***Note:***  In Jiménez et al. the SCWT - interference score was used as a measure of executive function.

**Sexual abuse**

***Note:*** In Jimenez et al. the SCWT - interference score was used as a measure of executive function.

**Emotional neglect**

**Physical neglect**

***Note:***  In Jimenez et al. the SCWT - interference score was used as a measure of executive function.

# **SA8. Qualitative synthesis of cognitive domains and studies not providing sufficient data for meta-analysis**

Two studies^15,16^ evaluated visual memory using the Rey–Osterrieth complex figure Test (ROCF)^17^ showing high CM was related to lower visual memory in people with BD. One study^18^ explored associations between CM and non-verbal memory using the CANTAB spatial recognition memory percent correct, CANTAB pattern recognition memory percent correct, and CANTAB paired associate learning total errors adjusted score, but no significant associations were found. One study^16^ explored fine motor dexterity including three subtest scores of the Purdue Pegboard (completion time for dominant, non-dominant, and both hands)^19^. One study^20^ evaluated orientation, language, and visuospatial abilities using the Addenbrooke's Cognitive Examination – revised (ACER-R)^21^, while other study evaluated visual copying using the Visual Copying Test of the Wechsler Memory Scale-Revised (WMS-R)^22^, but no associations between these cognitive domains and CM in BD were found.

Three of the 20 included studies explored cognitive functions that were not meta-analysed due to not enough data. One study^23^ found that impaired meta-cognition was associated with sexual abuse exposure in BD. Another study^24^ found that whilst CM was positively associated with current inter-episode depressive mood, no associations were found with BD severity or autobiographical memory specificity. Moreover, the association between CM and depressed mood was not moderated by autobiographical memory specificity. One study^25^ of moral decision-making found that higher ratings of physical neglect were associated with higher ratings of acceptability (a utilitarian tendency) across dilemma types, with a similar pattern being observed.

# **SA9. Meta-regression analyses**

Two moderating effects were found with sample size and study quality each decreasing the association between sexual abuse and verbal memory/learning (*β =* -.001; 95% CI [-0.002; -0,000], *p* = .010) and overall CM and social cognition (*β = -*.138; 95% CI [-0.251; -0,024], *p* = .017), and a moderating effect with study quality increasing the association between emotional abuse and executive functions/verbal fluency (*β =* .178; 95% CI [0.078; -0,277], *p* = .001). Also, associations between emotional abuse (*β = -*.031; 95% CI [-0.046; -0,017], *p* <.001), physical abuse (*β = -*.017; 95% CI [-0.025; -0,008], *p* = .0002), and emotional neglect (*β = -*.017; 95% CI [-0.031; -0,002], *p* = .023) and social cognition were weaker in males.

# **SA10. One-study-removed sensitivity analyses**

To further assess possible causes of heterogeneity and robustness of findings, one-study-removed sensitivity analyses were conducted. Removal of single effect sizes did not change the patterns of most results with a few exceptions: For the association between Overall CM and attention and processing speed, the removal of Ehrlich et al. (*r =* -0.193 [95% CI = -0.399, 0.032], *p* = 0.092) and Miskowiak et al. (*r =* -0.207 [95% CI = -0.399, 0.002], *p* = 0.052) led to not significant association, which was significant (negative) with the inclusion of these studies. For the association between overall CM and working memory the removal of Quidé et al. (*r =* -0.146 [95% CI = -0.232, -0.058], *p* < 0.001) led to significant negative association, which was not observed with the inclusion of this study. For the association between emotional abuse and executive functions and verbal fluency, the removal of Miskowiak et al. (*r =* -0.172 [95% CI = -0.326, -0.010], *p* = 0.038) led to significant negative associations which was not observed with the inclusion of this study. Finally, for the associations of overall CM (*r =* -0.360 [95% CI = -0.508, -0.191], *p* < 0.001), emotional neglect (*r =* -0.238 [95% CI = -0.400, -0.061], *p* = 0.009), and physical neglect (*r =* -0.242 [95% CI = -0.359, -0.116], *p* < 0.001) with social cognition, the removal of Morán-Kneer et al. led to negative associations which were not observed with the inclusion of this study.

# **SA11. Narrative synthesis of moderators and mediators reported in the included studies**

Seven of the included studies^5,15,24,26–30^ investigated effect moderation and one study^31^ investigated effect mediation between CM and cognitive outcomes.

One study^30^ examined moderating effects of lifetime childhood physical and sexual abuse and cognitive disruptions (sustained attention, affective information processing), on longitudinal ratings of depression symptom severity in youths. A significant moderating effect was detected for sustained attention and maltreatment history. In the context of lower sustained attention, maltreatment exposure was associated with higher depression symptom severity during childhood (<12 years), but not late adolescence.

Another study^24^ found that whilst CM predicted current inter-episode depressive mood, CM was not predictive of BD severity or autobiographical memory (including episodic and semantic memory components) specificity. Moreover, the association between CM and depressed mood was not moderated by autobiographical memory specificity.

One study^5^ found that the type of BD did not modify the associations between CM and poorer cognition across domains. Although one study proposed estimated IQ may act as a protective factor against cognitive decline in survivors with BD^15^, other study^5^ did not support moderation effect of verbal intelligence between CM and cognition. However, there is some evidence for associations between CM and impairment across global cognition and cognitive domains being mediated by lower general IQ (verbal and performance tasks) as measured by the WASI^31^. In addition, a study found that CM and current use of lithium and antipsychotic medication did not affect the relationship between changes in brain volumes and lower IQ^27^.

One study^26^ supported a moderating role of attachment anxiety but not attachment avoidance in the association between CM and social cognition. The authors found that in individuals with BD, higher attachment anxiety scores (but not avoidance anxiety) correlated with better performance in social cognition, suggesting that attachment anxiety may serve as an adaptive strategy for individuals with BD who experienced CM.

Another study found that CM-exposed individuals with BD type I showed deﬁcits speciﬁcally in social cognition, but not general cognitive abilities, and that increased schizotypy levels, especially paranoid ideation (suspiciousness), interacted with CM to predict altered social cognition skills, including ToM but not facial emotion processing^28^.

Finally, emerging evidence from one study points to interactive (moderating) effects of having experienced physical and emotional abuse in childhood and the oxytocin receptor gene on social cognition alterations, specifically in emotion recognition, in individuals with BD type I who were carriers of the GG genotype at *OXTR rs53576* ^29^.

# **ST1. PRISMA 2020 statement and checklist**

| **Section and Topic** | **Item #** | **Checklist item** | **Location where item is reported/ page** |
| --- | --- | --- | --- |
| **TITLE** | | |  |
| Title | 1 | Identify the report as a systematic review. | Cover page / 0 |
| **ABSTRACT** | | |  |
| Abstract | 2 | See the PRISMA 2020 for Abstracts checklist. | Abstract / 2 |
| **INTRODUCTION** | | |  |
| Rationale | 3 | Describe the rationale for the review in the context of existing knowledge. | Introduction/ 4-6 |
| Objectives | 4 | Provide an explicit statement of the objective(s) or question(s) the review addresses. | Introduction/ 6 |
| **METHODS** | | |  |
| Eligibility criteria | 5 | Specify the inclusion and exclusion criteria for the review and how studies were grouped for the syntheses. | Methods / 7 |
| Information sources | 6 | Specify all databases, registers, websites, organisations, reference lists and other sources searched or consulted to identify studies. Specify the date when each source was last searched or consulted. | Methods / 7 |
| Search strategy | 7 | Present the full search strategies for all databases, registers, and websites, including any filters and limits used. | Methods/ 6-7  Supplement SA2 |
| Selection process | 8 | Specify the methods used to decide whether a study met the inclusion criteria of the review, including how many reviewers screened each record and each report retrieved, whether they worked independently, and if applicable, details of automation tools used in the process. | Methods / 7 |
| Data collection process | 9 | Specify the methods used to collect data from reports, including how many reviewers collected data from each report, whether they worked independently, any processes for obtaining or confirming data from study investigators, and if applicable, details of automation tools used in the process. | Methods/ 7-9 |
| Data items | 10a | List and define all outcomes for which data were sought. Specify whether all results that were compatible with each outcome domain in each study were sought (e.g., for all measures, time points, analyses), and if not, the methods used to decide which results to collect. | Methods/ 8  Supplement SA3 |
|  | 10b | List and define all other variables for which data were sought (e.g., participant and intervention characteristics, funding sources). Describe any assumptions made about any missing or unclear information. | Methods /9-10 Supplement SA4 |
| Study risk of bias assessment | 11 | Specify the methods used to assess risk of bias in the included studies, including details of the tool(s) used, how many reviewers assessed each study and whether they worked independently, and if applicable, details of automation tools used in the process. | Methods/ 11, 14-15  Supplement ST6 |
| Effect measures | 12 | Specify for each outcome the effect measure(s) (e.g., risk ratio, mean difference) used in the synthesis or presentation of results. | Methods/ 9 |
| Synthesis methods | 13a | Describe the processes used to decide which studies were eligible for each synthesis (e.g., tabulating the study intervention characteristics and comparing against the planned groups for each synthesis (item #5)). | Methods/ 10 |
|  | 13b | Describe any methods required to prepare the data for presentation or synthesis, such as handling of missing summary statistics, or data conversions. | Methods/ 10-11 |
|  | 13c | Describe any methods used to tabulate or visually display results of individual studies and syntheses. | Methods/ 11 |
|  | 13d | Describe any methods used to synthesise results and provide a rationale for the choice(s). If meta-analysis was performed, describe the model(s), method(s) to identify the presence and extent of statistical heterogeneity, and software package(s) used. | Methods/ 10-12 |
|  | 13e | Describe any methods used to explore possible causes of heterogeneity among study results (e.g., subgroup analysis, meta-regression). | Methods/ 11 |
|  | 13f | Describe any sensitivity analyses conducted to assess robustness of the synthesised results. | Methods/ 11 |
| Reporting bias assessment | 14 | Describe any methods used to assess risk of bias due to missing results in a synthesis (arising from reporting biases). | Methods/ 11 |
| Certainty assessment | 15 | Describe any methods used to assess certainty (or confidence) in the body of evidence for an outcome. | Methods/11 |
| **RESULTS** | | |  |
| Study selection | 16a | Describe the results of the search and selection process, from the number of records identified in the search to the number of studies included in the review, ideally using a flow diagram. | Figure 1 Results/ 12 |
|  | 16b | Cite studies that might appear to meet the inclusion criteria, but which were excluded, and explain why they were excluded. | Results/ 12  Supplement |
| Study characteristics | 17 | Cite each included study and present its characteristics. | Results /12  Supplement SA6 |
| Risk of bias in studies | 18 | Present assessments of risk of bias for each included study. | Results/ 12 Supplement SA5 |
| Results of individual studies | 19 | For all outcomes, present, for each study: (a) summary statistics for each group (where appropriate) and (b) an effect estimates and its precision (e.g., confidence/credible interval), ideally using structured tables or plots. | Results/ 12-19  Table 1 |
| Results of syntheses | 20a | For each synthesis, briefly summarise the characteristics and risk of bias among contributing studies. | Results/ 14 Supplement ST6 |
|  | 20b | Present results of all statistical syntheses conducted. If meta-analysis was done, present for each the summary estimate and its precision (e.g., confidence/credible interval) and measures of statistical heterogeneity. If comparing groups, describe the direction of the effect. | Results Table 2  Figure 2  Supplement SA7 |
|  | 20c | Present results of all investigations of possible causes of heterogeneity among study results. | Results/ 20  exploratory  (n < 10) |
|  | 20d | Present results of all sensitivity analyses conducted to assess the robustness of the synthesised results. | Results/ 21  Supplement SA8 |
| Reporting biases | 21 | Present assessments of risk of bias due to missing results (arising from reporting biases) for each synthesis assessed. | NA (n < 10) |
| Certainty of evidence | 22 | Present assessments of certainty (or confidence) in the body of evidence for each outcome assessed. | Results/ 19-21  Supplement SA7, SA8,  ST6 |
| **DISCUSSION** | | |  |
| Discussion | 23a | Provide a general interpretation of the results in the context of other evidence. | Discussion/ 23 |
|  | 23b | Discuss any limitations of the evidence included in the review. | Discussion/ 23-24  Table 3 |
|  | 23c | Discuss any limitations of the review processes used. | Discussion/ 26 |
|  | 23d | Discuss implications of the results for practice, policy, and future research. | Discussion 25/26 |
| **OTHER INFORMATION** | | |  |
| Registration and protocol | 24a | Provide registration information for the review, including register name and registration number, or state that the review was not registered. | Abstract/ 2  Methods/ 6 |
|  | 24b | Indicate where the review protocol can be accessed, or state that a protocol was not prepared. | Abstract/ 2  Methods/ 6 |
|  | 24c | Describe and explain any amendments to information provided at registration or in the protocol. | NA  (no amendments) |
| Support | 25 | Describe sources of financial or non-financial support for the review, and the role of the funders or sponsors in the review. | Funding  Cover/ 1 |
| Competing interests | 26 | Declare any competing interests of review authors. | Competing interest declaration/1 |
| Availability of data, code and other materials | 27 | Report which of the following are publicly available and where they can be found: template data collection forms; data extracted from included studies; data used for all analyses; analytic code; any other materials used in the review. | Data availability statement/ 1 |

# **ST2. PRISMA 2020 for abstracts checklist**

| **Section and Topic** | **Item #** | **Checklist item** | **Reported (Yes/No)** |
| --- | --- | --- | --- |
| **TITLE** | | |  |
| Title | 1 | Identify the report as a systematic review. | Yes |
| **BACKGROUND** | | |  |
| Objectives | 2 | Provide an explicit statement of the main objective(s) or question(s) the review addresses. | Yes |
| **METHODS** | | |  |
| Eligibility criteria | 3 | Specify the inclusion and exclusion criteria for the review. | Yes |
| Information sources | 4 | Specify the information sources (e.g., databases, registers) used to identify studies and the date when each was last searched. | Yes |
| Risk of bias | 5 | Specify the methods used to assess risk of bias in the included studies. | Yes |
| Synthesis of results | 6 | Specify the methods used to present and synthesise results. | Yes |
| **RESULTS** | | |  |
| Included studies | 7 | Give the total number of included studies and participants and summarise relevant characteristics of studies. | Yes |
| Synthesis of results | 8 | Present results for main outcomes, preferably indicating the number of included studies and participants for each. If meta-analysis was done, report the summary estimate and confidence/credible interval. If comparing groups, indicate the direction of the effect (i.e. which group is favoured). | Yes |
| **DISCUSSION** | | |  |
| Limitations of evidence | 9 | Provide a brief summary of the limitations of the evidence included in the review (e.g. study risk of bias, inconsistency and imprecision). | Yes |
| Interpretation | 10 | Provide a general interpretation of the results and important implications. | Yes |
| **OTHER** | | |  |
| Funding | 11 | Specify the primary source of funding for the review. | Yes |
| Registration | 12 | Provide the register name and registration number. | Yes |

# **ST3. MOOSE guideline and checklist**

| **Criteria** | | **Brief description of how the criteria were handled in the meta-analysis** |
| --- | --- | --- |
| **Reporting of background should include** | |  |
| √ | Problem definition | To study them magnitude and consistency of associations between overall childhood maltreatment and its subtypes and cognitive functioning (global and its domains) in individuals with bipolar disorder, and to examine mediators and moderators in these relationships. |
| √ | Hypothesis statement | We hypothesised that there is a negative association between childhood maltreatment and cognitive functioning so that childhood maltreatment would be related to poorer cognitive outcomes in individuals with bipolar disorder. |
| √ | Description of study outcomes | Cognitive functioning: Global, Attention/Processing Speed, Verbal/Visual Memory and Learning, Working Memory, Executive Functions/Verbal Fluency, Social Cognition. |
| √ | Type of exposure or intervention used | Studies included were original articles investigating individuals with bipolar disorders exposed to childhood maltreatment (no intervention). |
| √ | Type of study designs used | Cross-sectional and longitudinal studies (only with baseline data). |
| √ | Study population | Individuals with bipolar disorders (Type I and II) defined according to international and established criteria (DSM, ICD). |
| **Reporting of search strategy should include** | |  |
| √ | Qualifications of searchers | The credentials of the investigators/reviewers are indicated in the author list and in the acknowledgements. |
| √ | Search strategy, including time period included in the synthesis and keywords | We used specific keywords relative to the type of the diverse cognitive functioning domains, child maltreatment, and diagnoses of interest (bipolar disorders). A second step involved manual search of the reference lists of the retrieved articles. |
| √ | Databases and registries searched | PsycINFO, Embase, PubMed (Medline), Web of Science (core collection), Cochrane, and PILOTS. |
| √ | Search software used, name and version, including special features | Zotero 5.0.96.3, Ryann |
| √ | Use of hand searching | We hand-searched bibliographies of retrieved papers for additional references. |
| √ | List of citations located and those excluded, including justifications | Details of the literature search process are outlined in the PRISMA flowchart. |
| √ | Method of addressing articles published in languages other than English | No language or date limits were applied. The search included abstract in any language. |
| √ | Method of handling abstracts and unpublished studies | Abstracts and unpublished studies (grey literature) were excluded. |
| √ | Description of any contact with authors | We contacted all the corresponding authors to provide additional data for the meta-analysis when needed (at least twice). |
| **Reporting of methods should include** | |  |
| √ | Description of relevance or appropriateness of studies assembled for assessing the hypothesis to be tested | Detailed inclusion and exclusion criteria were described in the methods section. |
| √ | Rationale for the selection and coding of data | Data extracted from each of the studies were related to the population characteristics, study design, exposure, outcome, and effect of confounders, mediators and moderators reported in the included studies. |
| √ | Assessment of confounding | Meta-regressions were used to examine the quantitative influence of several predefined variables and quality of studies. Additionally, we qualitatively estimate the influence of confounders in the association between childhood maltreatment and cognitive functioning in bipolar disorder. |
| √ | Assessment of study quality, including blinding of quality assessors; stratification or regression on possible predictors of study results | We adapted the Newcastle-Ottawa Scale for the evaluation of non-randomised studies. This tool has been adopted in recent meta-analyses^2,3^. |
| √ | Assessment of heterogeneity | Heterogeneity was assessed using the *Q*-test and *I^2^* index. |
| √ | Description of statistical methods in sufficient detail to be replicated | Random effect meta-analyses. Description of methods of meta-analyses, sensitivity analyses, meta-regressions and assessment of publication bias were fully detailed in the methods. |
| √ | Provision of appropriate tables and graphics | We included the PRISMA flow-chart and several tables to describe the literature search and its results. Several tables and forest/funnel plots, and figures were used to describe the main findings of the analyses. |
| **Reporting of results should include** | |  |
| √ | Graph summarising individual study estimates and overall estimate | We have appended illustrative table 2 in the main text. Additional tables were presented as supplementary material to fully describe the results. |
| √ | Table giving descriptive information for each study included | Table 1. |
| √ | Results of sensitivity testing | Sensitivity analyses (one-study-removal) were reported in the main text and plots were appended in the supplementary results. |
| √ | Indication of statistical uncertainty of findings | We did report mean estimates for the main outcome, pooled 95% CI, and prediction intervals. |
| **Reporting of discussion should include** | |  |
| √ | Quantitative assessment of bias | Descriptions of quantitative assessment of bias are detailed in the methods; results are described in the main text, and funnel plots are appended in the supplementary materials. |
| √ | Justification for exclusion | Exclusion criteria about publication type, language, diagnosis definition of bipolar disorder, measures and instruments for childhood maltreatment and cognitive functioning, the presence of treatment outcomes, a relationship between childhood maltreatment and cognitive functioning in bipolar disorder (or descriptive statistics that allows calculating associations) reported, or any meta-analytical data provided, are detailed in methods, while discussed in the discussion. |
| √ | Assessment of quality of included studies | The assessment of quality of included studies, using the Newcastle-Ottawa Quality Assessment Scale is detailed in the supplement, and results of the quality assessment, that we entered in meta-regression analyses are discussed in the discussion. |
| **Reporting of conclusions should include** | |  |
| √ | Consideration of alternative explanations for observed results | We discussed alternative explanations for our findings, specifically considering potential methodological shortcomings. |
| √ | Generalisation of the conclusions | We have clearly addressed the generalisation of the conclusions in the discussion section. |
| √ | Guidelines for future research | We have suggested possible streams of future research in the discussion. |
| √ | Disclosure of funding source | We added a funding disclosure for the undertaking of this systematic review and meta-analysis. |

# **ST4. Manuals codes for bipolar disorder diagnoses included**

| **Diagnosis** | **Codes used within ICD-10** | **Codes used within ICD-11** | **Codes used within DSM-IV** | **Codes used within DSM-5** |
| --- | --- | --- | --- | --- |
| Bipolar disorder type I | F31.0, F31.1x, F31.2, F31.3x, F31.4, F31.5, F31.6, F31.7x, F31.9 | 6A60 | 296.4x, 296.5x, 296.6x, 296.7 | 296.4x, 296.5x, 296.7 |
| Bipolar disorder type II | F31.81 | 6A61 | 296.89 | 296.89 |
| Bipolar disorder not otherwise specified | F31.9 | 6A6Z | 296.80 | 296.80 |

# **ST5. Description and measurement of cognitive functioning**

| **Cognitive Functioning** | |
| --- | --- |
| **Global cognition and IQ** | |
| ***Definition****:* Global cognition and IQ domain reflects a measure of general cognitive ability capacities, integrating memory, attention, executive function, language, and visuospatial skills. The IQ typically presents a composite score that summarises performance across diverse cognitive tasks. | |
| **Instrument / Measurement** | **Description** |
| Addenbrooke's Cognitive Examination Revised (ACE-R), Chilean version | The ACE-R is a brief cognitive test that assesses five cognitive domains, namely attention/orientation, memory, verbal fluency, language and visuospatial abilities. Total score is 100, higher scores indicates better cognitive functioning. Administration of the ACE-R takes on average 15 minutes^21^. |
| Brief Assessment of Cognition in Affective Disorders (BAC-A) | The BAC-A is a general tool to assess cognitive performance in patients with affective disorders, including six neurocognitive domains: verbal memory (list learning), motor speed (token motor task), working memory (digit sequencing task), verbal fluency (category instances and controlled oral word associations test), processing speed (symbol coding) and executive functions (tower of London). A composite score is a *Z*-score derived from all six subtests based on a norm reference^32^. |
| Kaufman Brief Intelligence Test (K-BIT) | The K-BIT is a brief intelligence assessment scale which measures verbal (fluid) and non-verbal (crystallised) intelligence domains, providing a composite value in a IQ-scale^33^. |
| National Adult Reading Test, original, Danish and Dutch versions full-scale IQ (NART)^34,35^ | The NART measures verbal and premorbid IQ. Participants are asked to read out loud irregular words^34^. |
| Repeatable Battery for the Assessment of Neuropsychological Status (RBANS) | The RBANS is a general cognitive assessment tool, which contains 12 subtests which makes up 5 age-adjusted indexes: immediate memory, visuospatial, language, attention, and delayed memory^36^. |
| Wechsler Abbreviated Scale of Intelligence (WASI) | The WASI is a short intelligence assessment, derived from Wechsler Adult Intelligence Scale (WAIS), composed of four subtests: Vocabulary (verbal abilities); Similarities (verbal abilities); Block Design (perception and visuo-spatial abilities); and Matrix Reasoning (perception and visuo-spatial abilities)^37^. |
| Wechsler Adult Intelligence Scale (WAIS), Adult Reading Test subscale | The Adult Reading subtest from the WAIS is used to estimate crystallised intelligence, and to measure premorbid IQ^38^. |
| Wechsler Adult Intelligence Scale-III (WAIS-III), vocabulary subtest | The WAIS-III - vocabulary subtest is used to estimate premorbid IQ. Individuals are asked to define words that range from very common to more obscure. The participant's responses are scored based on accuracy and completeness, with higher scores indicating better performance. This subtest usually takes about 10 to 15 minutes to complete^39^. |
| WAIS-III | The WAIS-III scale consists of several tasks designed to assess different cognitive abilities: 1) Verbal Comprehension, including vocabulary and similarities to measure verbal abilities; 2) Perceptual Organisation, including block design and matrix reasoning tasks to assess visual and spatial skills; 3) Working Memory, including digit span and arithmetic tasks to evaluate short-term memory and attention; and 4) Processing speed including symbol search and coding. Scores from these subtests are combined to give an overall IQ score. The WAIS-III takes around 60 to 90 minutes to complete^39^. |
| **Neurocognition** | |
| **Attention and processing speed** | |
| ***Definition****:* Attention encompasses various subcomponents, including sustained attention (the ability to maintain focus over prolonged periods), selective attention (the ability to focus on relevant stimuli while filtering out distractions), and divided attention (the capacity to process multiple sources of information simultaneously)^2^ and processing speed is the rate at which an individual can identify, integrate, and respond quickly to visual or verbal information^3^. | |
| **Instrument / Measurement** | **Description** |
| Cambridge Neuropsychological Test Automated Battery, Rapid Visual Information Processing test (RVIP), A’ score, latency and discriminability score^40^ | The RVIP test assesses sustained attention and is analogous to the well-known Continuous Performance Test (CPT). A white box appears on the screen and a series of numbers (digits 2–9) appear in the middle of the box in a pseudo-random order at a rate of 100 digits per minute. Participants are instructed to hit a button when they detect a target sequence of numbers (e.g., every time they see the sequence “2–5–9″). The target sequence varies in difficulty from one to three digits. Outcome measures in the present analysis included total hits (accuracy), total misses (errors), and A′, a signal detection theory measure of sensitivity to errors^41^. |
| Continuous Performance Test-II (CPT-II) computerised version | The CPT-II measures attention and response control. Participants watch a screen and press a button whenever a specific letter appears, while avoiding pressing for other letters. The test tracks reaction times and accuracy, with errors and response times providing the scores. The CPT-II usually takes about 14 minutes to be completed^42^. |
| Repeatable Battery for the Assessment of Neuropsychological Status (RBANS), Digit span forward subtest, correct answers | The Digit Span Forward subtest in the RBANS assesses short-term memory and attention. In this task, the participant hears a sequence of numbers and must repeat them in the same order. Scoring is based on how many sequences are correctly repeated, with higher scores showing better performance. This subtest usually takes about 5 minutes to be completed^36^. |
| RBANS, Coding subtest, correct answers | The RBANS’ Coding subtest assesses processing speed and visual-motor coordination. In this task, participants match symbols with corresponding numbers as quickly as they can within a time limit. Scoring is based on the number of correct matches made in the given time. The subtest typically takes around 2 minutes to be completed^36^. |
| Screen for Cognitive Impairment in Psychiatry (SCIP), psychomotor speed composite score | In the SCIP participants perform a digit substitution task. The completion time is recorded, with quicker times indicating better performance. This subtest usually takes about 30 seconds to be administered^43^. |
| Trail Making Test, Part A (TMT-A), total time to completion | The TMT-A is a simple, quick assessment often used to measure cognitive processing speed and visual attention. In Part A, the participant is asked to draw lines to connect consecutively numbered circles (1, 2, 3, etc.) scattered on a page as quickly as possible. Scoring is based on the time it takes to complete the task, with faster times indicating better performance. The test typically takes about 1 minutes to be administered^44^. |
| WAIS-III, Symbol search and Digit-Symbol Coding subtests | The Wechsler Adult Intelligence Scale-III test includes the Symbol Search and Digit-Symbol Coding subtests to evaluate processing speed. In Symbol Search, participants look at symbols and determine if they match a target symbol within a time limit. For Digit-Symbol Coding, they pair numbers with corresponding symbols based on a key. Both tasks are scored on the number of correct responses within the time allowed, typically taking about 2 minutes each to be completed^39^. |
| **Verbal memory and learning** | |
| ***Definition:*** verbal memory involves encoding, storage, and retrieval of language-based material like words, sentences, and stories^4^ typically assessed using tasks that require recall or recognition of verbal stimuli. Learning, closely related to memory, is the process through which individuals acquire new information or skills through experience, practice, or instruction^5^. | |
| **Instrument / Measurement** | **Description** |
| Autobiographical Memory Test (AMT), specific, categoric, extended, non-interpretable and omission scoring categories | The AMT Test is used as a method of assessing autobiographical or personal event memory in people with emotional disturbance, including components of sematic and episodic memory. Participants were asked to retrieve specific AMTs in response to emotional cue words. Twelve cue words (6 positive and 6 negative) are used. Responses were audio recorded and subsequently rated by the experimenter. The following scoring categories were used: Specific; Categoric; Extended; Does not comply with task or non-interpretable; Omission^45,46^. |
| California Verbal Learning Test (CVLT), Second Edition, total learning five trials, short delay free recall, short delay cued recall, long delay free recall, long delay cued recall, and recognition hits | The CVLT is a tool used to assess verbal memory and learning. Participants hear a list of words and then recall as many as they can immediately and after a delay. Scores are based on the number of words correctly recalled. On average, the CVLT takes about 20 to 30 minutes to be administered^47^. |
| Cambridge Neuropsychological Test Automated Battery CANTAB), Paired Associate Learning (PAL), total errors adjusted score^40^ | The PAL test evaluates visual memory and learning ability. Participants are shown pairs of abstract visual stimuli in sequence and then asked to match each pair from memory. Scoring reflects both accuracy and the ability to learn new associations over trials. On average, this test takes about 8 minutes to be completed^48^. |
| CANTAB, Pattern Recognition Memory (PRM) | The PRM^40^ test in the CANTAB^40^ assesses visual memory and recognition. Participants view a series of abstract visual patterns presented on a screen. Later, they are shown pairs of patterns, one they've seen before and a new one, and they are asked to identify the pattern they previously viewed. Scoring is based on accuracy in recognising the previously viewed patterns. The average administration time for the PRM test is approximately 4 minutes^49^. |
| CANTAB, Spatial Recognition Memory (SRM) | The SRM assesses visual memory for spatial locations. Participants view a series of patterns on a screen and are asked to determine if each pattern matches one, they've seen before. Scoring is based on accuracy in recognising the correct matches^40^. |
| Rey-Auditory Verbal Learning Test (RAVLT), Trial I to V, Trial IV Delayed Recall Recognition, total correct answers^50^ | The RAVLT assesses verbal memory and learning. Participants listen to a list of words and then immediately recall as many as possible. This process is repeated over several trials. Scoring is based on the number of words remembered across different trials and how well they recall these words after a delay. The RAVLT typically takes about 15 to 20 minutes to be administered^50^. |
| Rey-Osterrieth Complex Figure Test (ROCF)^17^ | The ROCF is a neuropsychological assessment used to evaluate visuospatial abilities and visual memory, and immediate recall, delayed recall, and recognition in particular. Participants are asked to copy a complex geometric figure, which involves detailed visual processing and motor coordination. Scoring is based on accuracy and completeness of the figure copy, assessing spatial organisation and detail orientation. Additionally, participants may be asked to recall and reproduce the figure from memory after a delay, which evaluates visual memory skills. Administration of the complete ROCF typically takes about 30 minutes^17^. |
| Screen for Cognitive Impairment in Psychiatry (SCIP), verbal learning and delayed recall composite score | The SCIP includes verbal memory/learning and delayed recall subtests to assess memory function. In the verbal learning task, participants listen to and recall a list of words over several trials. They are scored based on how many words they remember correctly. For delayed recall, participants are asked to recall the same list of words after a delay period. These two tests take 5 minutes to be administered^43^. |
| Wechsler Memory Scale-Revised (WMS-R), Visual Copying Test (VCT), total score | The VCT measures visual memory and visuospatial skills and memory subdomains. Participants are instructed to copy a complex geometric figure shown to them. Scoring is based on accuracy and completeness of the copied figure, evaluating spatial perception and motor coordination. The average administration time for this test is approximately 10 minutes^22^. |
| **Working Memory** | |
| ***Definition:*** this cognitive system encompasses the ability to hold and process information over short periods, integrating new information with existing knowledge to guide decision-making and behaviour^6,7^. | |
| **Instrument / Measurement** | **Description** |
| Auditory Consonant Trigrams Test, Turkish version, total score | The Auditory Consonant Trigrams Test assesses working memory and attention. Participants listen to sequences of consonant trigrams and are then asked to recall them after a delay period. Scoring is based on the number of trigrams correctly recalled. The average administration time for this test is approximately 5 to 10 minutes^51^. |
| CANTAB, Spatial Working Memory (SWM) test, between error and strategy score^40^ | The SWM test measures spatial planning and working memory. Participants are asked to remember and recall the location of hidden tokens within an on-screen grid. Scoring reflects accuracy and strategy in token placement. On average, this test takes about 4 to 6 minutes to be administered^52^. |
| Screen for Cognitive Impairment in Psychiatry (SCIP), working memory composite score^43^ | The SCIP - working memory subtest assesses short-term memory and attention. Participants are presented with a series of tasks requiring them to remember and manipulate information under time constraints. Scoring reflects both accuracy and speed of task completion. This subtest typically takes 5 minutes to be completed^43^. |
| Wechsler Adult Intelligence Scale-III, Letter-Number-Sequencing (WAIS-III-LNS), correct answers^39^ | The WAIS-III-LNS evaluates working memory and mental flexibility. Participants are given sequences of numbers and letters to rearrange into specific orders based on given rules. Scoring is based on accuracy in sequencing within a time limit. The average administration time for this test is approximately 5 minutes^39^. |
| Wechsler Adult Intelligence Scale-III (WAIS-II), Working memory index, composite score^39^ | A composite score is obtained from three subtests of the WAIS-III: Arithmetic, Digits, and LNS^39^. |
| **Executive functions and verbal fluency** | |
| ***Definition:*** higher-order cognitive processes that enable goal-directed behaviour, problem-solving, and adaptive responses to novel or complex situations, including cognitive abilities such as planning, inhibitory control, cognitive flexibility, and task switching^4,8,9^. Verbal fluency is also an executive function skill defined as the capacity to generate words rapidly and efficiently within specific constraints, reflecting lexical retrieval, language production, and cognitive flexibility^5^. | |
| **Instrument / Measurement** | **Description** |
| Barrat Impulsiveness Scale (BIS) inhibitory control cognitive, no-planning subscales^53^ | The BIS is a 30-item self-rated scale designed to assess impulsive thought and behaviour. Previous analysis identified two factors from the extracted of the Brazilian version of the scale: Inhibition control, defined as the ability to inhibit a proponent behaviour and attentional control; and Non-planning, defined as the decision making that requires cost-benefit evaluation between short and long term consequences within an emotional context^54^. |
| CANTAB Affective Go/No-Go Task, commission errors (incorrect response to a distractor), omission errors (incorrect response to a target), and latency (milliseconds) to a correct response^40^ | The CANTAB Affective Go/No-Go Task assesses ‘hot’ inhibitory control, or information processing for negative and positive valence words. Across several blocks, a series of words is presented from two of three affective categories: (1) positive (e.g., joyful), (2) negative (e.g., hopeless), and (3) neutral (e.g., element). Participants are instructed to press a button (go) when they view a word that matches the target category (e.g., the word “happy” appears when the category is positive words) and withhold a response (no-go) when they view a word that does not match the target category (e.g., the word “despair” in the category of positive words). Outcome measures include commission errors (incorrect response to a distractor), omission errors (incorrect response to a target), and latency (milliseconds) to a correct response^40^. |
| CANTAB, Intra-/extra-dimensional (IED), set-shifting task number of extra-dimensional shifting errors: set-shifting and cognitive flexibility | The IED test assesses cognitive flexibility and attentional set-shifting. Participants learn and shift between rules to categorise visual stimuli based on feedback. Scoring reflects accuracy in adapting to new rules. The average administration time for this test is approximately 7 minutes^55^. |
| CANTAB, One Touch Stockings of Cambridge (OTS), mean choices to correct and mean latency to correct scores | The OTS test measures planning and problem-solving abilities. Participants are asked to move coloured balls in stockings to match a target pattern shown on the screen. Scoring is based on accuracy and efficiency of solving the problem. On average, this test takes about 10 minutes to complete^56^. |
| Controlled Oral Word Association Test (COWAT), FAS phonemic fluency, KAS phonemic fluency and categorical (animal) fluency tasks^57^ | The COWAT evaluates verbal fluency and executive function. Participants are instructed to generate words belonging to specific categories (e.g., animals) or phonemic families within a limited time frame. Scoring is based on the number of correct responses produced^57^. |
| Parametric Go/No-Go (PGNG), mean accuracy of target trials, mean target response time and mean accuracy for the inhibitory trials^58^ | The PGNG test assesses inhibitory control and set shifting including attention and executive functioning with three levels of increasing difficulty. Level 1 measures accuracy and response time to three targets. Level 2 adds a nonrepeating rule, measuring response time to two targets, accuracy for targets, and accuracy for appropriate inhibition. Level 3 has three targets with the same nonrepeating rule. The task shows good construct validity, and factor analyses show adequate ability to distinguish between processing speed, sustained attention, and inhibition. Normative data for the PGNG are stratified by age and education^58^. |
| Screen for Cognitive Impairment in Psychiatry (SCIP), verbal fluency composite score^43^ | The SCIP - verbal fluency subtest evaluates language and executive function. Participants are instructed to generate words beginning with specific letters within a limited time frame. Scoring is based on the number of correct responses. This subtest typically takes about 1 minute to be administered^43^. |
| Stroop Colour-Word Test (SCWT), Stroop word, Stroop colour and Stroop colour-word scores^59^ | The SCWT test measures inhibitory control, cognitive flexibility, and selective attention. Participants are presented with words printed in incongruent colours (e.g., the word "red" printed in blue ink) and are asked to name the ink colour while ignoring the word's meaning. Scoring is based on accuracy and speed of responses. The average administration time for this test is approximately 5 to 10 minutes^60^. |
| Trail Making Test (TMT), Part B, total time | Part B of the TMT assesses cognitive flexibility, visual attention, and task switching. Participants connect alternating letters and numbers in sequence (e.g., 1-A-2-B-3-C, etc.). Scoring reflects accuracy and completion time. The average administration time for this test is approximately under 3 minutes^44^. |
| Verbal fluency test, with letters S and D, total correct words | This test assesses verbal fluency and executive function. Participants are asked to generate words starting with the letter "S" and "D" within a time limit. Scoring is based on the number of appropriate words produced. On average, this test takes about 3 to 5 minutes to complete^61^. |
| Wisconsin Card-Sorting Test (WCST), completed categories and preservative errors scores^62^ | The computerised version of the WCST with a 64-card deck is used to assess cognitive flexibility and general executive functions. Patients are required to match response cards to four stimulus cards along one of the three dimensions (colour, form, or number) on the basis of sign feedback (correct or wrong). The subjects were not given any information about the dimensions. After sorting a series of 10 cards in one category, subjects were asked to sort the cards again in a different category. The indices of completed categories and preservative errors were used to assess performance in the test^63^. |
| **Social Cognition** | |
| ***Definition:*** Social cognition encompasses a range of processes, from perceiving and decoding social information to decision-making, which underlies our ability to "make sense of others' behavior"^10,^ including both basic abilities, such as face processing and joint attention, and more complex ones, like theory of mind, moral reasoning, and social decision-making^11^. | |
| **Instrument / Measurement** | **Description** |
| Mayer–Salovey–Caruso Emotional Intelligence Test (MSCEIT), version 2.0, Managing emotions task | The MSCEIT assesses emotional intelligence through tasks related to perceiving, using, understanding, and managing emotions. Participants respond to scenarios and answer questions based on emotional content. Scoring is based on accuracy in understanding emotional situations. The average administration time for this test is approximately 30 to 45 minutes^64^. |
| The Awareness of Social Inference Test (TASIT) | Participants completed all three parts of Form A. The ‘Emotion Evaluation Test’ (Part 1) comprises 28 short video clips (15–60 s) in which an actor portrays one of six basic emotions (happy, sad, fear, disgust, surprise, and anger); the assessment of ‘Social Inference – Minimal’ (Part 2) comprises 15 video clips (20–60 s) depicting sincere and sarcastic (simple sarcasm and paradoxical sarcasm) interactions between two actors, and at the end of each clip, participants answered four questions designed to elicit interpretations of what the speaker was thinking, doing (e.g., criticising), meaning to say, and feeling. The assessment of ‘Social Inference – Enriched’ (Part 3) comprises 16 vignettes (15–60 s) where participants are provided with extra (contextual) information about the true state of affairs before or after the dialogue of interest. Participants are examined on their ability to detect deception in social encounters (i.e., lies) and sarcasm. Administration time is 60–75 min, with a playing time of around 35 min^65^. |
| **Theory of Mind** | |
| ***Definition:*** the ability to reason about mental states and understand intentions, dispositions, emotions, and beliefs of both self and others. involves higher-order social cognitive processes to facilitate accurate understanding of others' thoughts, emotions, and intentions (i.e., mental states), which in turn facilitates adaptive social behaviour^66^. | |
| **Instrument / Measurement** | **Description** |
| Dokuz Eylul Theory of Mind (ToM) Index (DEZIKO)^67^ | DEZIKO assesses the ToM abilities using 16 questions. First-order false belief is measured with four questions, second-order false belief with three questions, irony with three questions, metaphor with two questions, and faux pas with one question^68^. |
| Hinting Task | The Hinting Task measures the ability to infer real intentions behind indirect speech utterances (mental state reasoning or ToM). The test is comprised of ten short passages presenting an interaction between two characters that ends with one of the characters dropping a hint. The subject is asked what the character really meant. Correctly identified hints were scored 2; in case of an incorrect response, a more obvious hint was added, and the subsequent correct response was scored 1; incorrect responses were scored 0. The outcome measure was the sum of the scores of the ten items (range 0–20)^69^. |
| **Emotion perception** | |
| **Definition:** the ability to identify and understand emotions of others and to manage emotions of oneself^70^. | |
| **Instrument / Measurement** | **Description** |
| Cambridge Neuropsychological Test Automated Battery, Emotion Recognition Task (ERT), correct answers and response latency^40^ | The ERT is a computer-generated paradigm for the recognition of six basic facial emotional expressions: happiness, sadness, anger, disgust, fear, and surprise. The emotions (15 stimuli for each emotion with different levels of intensity) are mimicked by actors and presented randomly in two blocks (90 stimuli each). After each stimulus presentation (200ms), the participant is asked to choose between the six emotional expressions displayed in labels on the screen. The task provides a percentage of correctness for each emotion and an overall mean of response latency^71^. |
| Emotion Perception Test (EPT), number or errors | The EPT measures the ability to recognise emotions from facial expressions, voice tone, and situational cues. Participants view images or listen to recordings and identify emotions portrayed. Scoring is based on accuracy in emotion recognition^72^. |
| Facial Expressions of Emotion- Stimuli and Tests | Static facial emotion perception (emotion processing). Participants are required to identify six basic emotions from the original Ekman series (happiness, sadness, anger, fear, surprise, and disgust) presented in a still photographic format^73^. |
| Test of Facial Recognition (TFR), accuracy and response time | The TFR assesses the ability to recognise and remember faces. Participants view images of faces and are asked to recall them after a delay. Scoring reflects accuracy in face recognition and memory. The average administration time for this test varies depending on the specific protocol used, typically ranging from 10 to 20 minutes^74^. |

# **ST6. Quality assessment of included studies**

Two independent reviewers (NEF-O, RL-E) used an adapted version of the Newcastle-Ottawa Scale (NOS) for non-randomised studies to assess the quality of included studies; discrepancies were resolved thorough consensus. Details on this scale can be found in the NOS scoring section in ST7. Studies were assessed based on three broad domains: 1) Selection of participants (representativeness of exposed samples; sample size, non-response rate or non-responders compared, and ascertainment of exposure; 2) Comparability involving appropriate control for confounders; 3) Assessment of outcome based on validated scales, and statistical analysis clearly described and allowing to calculate associations.

The maximum number of scores for each domain was 4 for Selection, 2 for Comparability, and 2 for Outcome. Total quality scores ranged from 0 to 8, with a higher score representing better quality. We rated the overall quality according to specific combinations of results across the three domains, according to the scale adaptation for cross-sectional studies. Studies scoring 5 points, that is, 2 points in selection, 1 point in comparability, and 2 points in outcome were considered as “fair” quality. Studies scoring 6, that is, 3 points in Selection, 1 point in Comparability, and 2 points in Outcome were classified as “good” quality. “High” quality studies scored > 6, that is, 3 or 4 points in Selection, 1 or 2 points in Comparability, and 2 points in Outcome (see the NOS scoring, and the results of the quality assessment of the included studies below).

Across the included studies, the representativeness of samples was mixed. Most of the studies included a satisfactory sample size, reported a priori power analyses or justified their sample sizes, but not reported on non-response. Most of the included studies controlled for confounders in their design or analysis, and adjusted for sex or gender, or any additional factor such as age, education level, and mood symptoms (see Table 1). All studies used standard or validated tools to assess CM and cognitive functioning. Some studies did not fully report results from statistical tests, e.g., omitting named effect estimates, or measures of precision if appropriate (such as standard errors or CIs).

## **Newcastle-Ottawa scale for non-randomised studies**

***Selection (maximum 4 stars)***

**1. Representativeness of the sample**

a. Truly representative of the average in the target population (all subjects or whole population, random sampling). *

b. Somewhat representative of the average in the target population (evidence that the sample is representative of the source population, non-random sampling). *

c. Selected group of users.

d. No description of the sampling strategy.

**2. Sample size**

a. Justified or satisfactory. *

b. Not justified.

**3. Non-respondents**

a. Comparability between respondents and non-respondents characteristics is established, or the response rate is satisfactory (> 60%) *

b. The response rate is unsatisfactory, or the comparability between respondents and non-respondents is unsatisfactory.

c. No description of the response rate or the characteristics of the responders and non-responders.

**4. Ascertainment of the exposure** (childhood maltreatment)

a. Validated measurement tool. *

b. Non-validated measurement tool, or not described.

***Comparability (maximum 2 stars)***

**5. The subjects in different outcome groups are comparable, based on the study design** **or analysis** (confounding factors are controlled).

a. The study controls for sex/gender (or analysis separated by sex/gender). *

b. Study controls for any additional factor. *

c. The study does not adjust for any confounding factor.

***Outcome (maximum 2 stars)***

**6. Assessment of the outcome** (social functioning)

a. The study uses reliable and validated instruments as confirmed by reported psychometric measures (behavioural tasks or questionnaires). *

b. No description.

**7. Statistical test**

a. The statistical test used to analyse the data is clearly described and appropriate, and the measurement of the association is presented, including confidence intervals and the probability level (p value). *

b. Study reports descriptive statistics that allows calculating associations. *

c. The statistical test is not appropriate, not described or incomplete.

***Note:***  In the Newcastle-Ottawa Scale (NOS) ^75^ adapted and validated ^76–79^ for non-randomised studies (cross-sectional and longitudinal)*,* a study can be awarded a maximum of one point (star) for each numbered item with the Selection and Exposure categories. A maximum of two points (stars) can be given for Comparability (see the quality assessment score below).

## **Quality assessment score**

| Newcastle-Ottawa Scale Criteria  *Cross-sectional studies* | **Maximum score = 8** |
| --- | --- |
| **Selection** | **4** |
| Sample representative of target sample (e.g., all eligible or random sample)? | 1 |
| Sample size justified or satisfactory? | 1 |
| Non-response rate is defined satisfactory, and characteristics of responders/non-responders compared? | 1 |
| Ascertainment of exposure (i.e., childhood maltreatment) is valid and/or well described? | 1 |
| **Comparability** | **2** |
| Controls for sex or gender | 1 |
| Controls for any additional factor | 1 |
| **Outcome** | **2** |
| Assessment of outcome with standardised or validated tool? | 1 |
| Statistical test clearly described and appropriate, and/or descriptive statistics that allows calculating associations? | 1 |

## **Results of the study quality assessment of the included studies**

| **Authors and year** | **1** | **2** | **3** | **4** | **5** | **6** | **7** | **TOTAL** |
| --- | --- | --- | --- | --- | --- | --- | --- | --- |
| Aas et al. 2012 | 1b* | 2a* | 3c | 4a* | 5a*+5b* | 6a* | 7a* | 7 |
| Arat-Çelik et al. 2023 | 1b* | 2a* | 3c | 4a* | 5a*+5b* | 6a* | 7a* | 7 |
| Bücker et al. 2013 | 1c | 2a* | 3c | 4a* | 5b* | 6a* | 7a* | 5 |
| Ehrlich et al. 2023 | 1b* | 2a* | 3c | 4a* | 5a*+5b* | 6a* | 7b* | 7 |
| Hsieh et al. 2021 | 1b* | 2b | 3c | 4a* | 5b* | 6a* | 7c | 4 |
| Jiménez et al. 2017 | 1b* | 2a* | 3c | 4a* | 5a*+5b* | 6a* | 7b* | 7 |
| Larsen et al. 2019 | 1b* | 2a* | 3c | 4a* | 5a*+5b* | 6a* | 7b* | 7 |
| Lima et al. 2017 | 1b* | 2a* | 3c | 4a* | 5b* | 6a* | 7a* | 6 |
| Martins et al. 2019 | 1b* | 2a* | 3c | 4a* | 5b* | 6a* | 7a* | 6 |
| Miskowiak et al. 2023 | 1b* | 2a* | 3a* | 4a* | 5a*+5b* | 6a* | 7a* | 8 |
| Morán-Kneer et al. 2022 | 1c | 2a* | 3c | 4a* | 5c | 6a* | 7a* | 4 |
| Mowlds et al. 2010 | 1a* | 2a* | 3a* | 4a* | 5b* | 6a* | 7a* | 7 |
| Oymak-Yenilmez et al. 2021 | 1c | 2a* | 3c | 4a* | 5b* | 6a* | 7a* | 5 |
| Quidé et al. 2018 | 1c | 2a* | 3c | 4a* | 5a*+5b* | 6a* | 7b* | 6 |
| Ríos et al. 2020 | 1c | 2a* | 3c | 4a* | 5a*+5b* | 6a* | 7b* | 6 |
| Ríos et al. 2023 | 1c | 2a* | 3c | 4a* | 5a*+5b* | 6a* | 7b* | 6 |
| Russo et al. 2015 | 1b* | 2a* | 3c | 4a* | 5b* | 6a* | 7b* | 6 |
| Takim et al. 2024 | 1c | 2a* | 3c | 4a* | 5a*+5b* | 6a* | 7a* | 6 |
| Vaughn-Coaxum et al. 2021 | 1b* | 2a* | 3a* | 4b | 5a*+5b* | 6a* | 7b* | 7 |
| Vreeker et al. 2017 | 1c | 2b | 3a* | 4a* | 5a*+5b* | 6a* | 7a* | 6 |

# **REFERENCES**

1. Fares-Otero NE, Seedat S. Childhood maltreatment: A call for a standardised definition and applied framework. *Eur Neuropsychopharmacol*. 2024;87:24-26. doi:10.1016/j.euroneuro.2024.07.002

2. Fares-Otero NE, De Prisco M, Oliva V, et al. Association between childhood maltreatment and social functioning in individuals with affective disorders: A systematic review and meta-analysis. *Acta Psychiatr Scand*. 2023;148(2):142-164. doi:10.1111/acps.13557

3. Fares-Otero NE, Alameda L, Pfaltz MC, Martinez-Aran A, Schäfer I, Vieta E. Examining associations, moderators and mediators between childhood maltreatment, social functioning, and social cognition in psychotic disorders: a systematic review and meta-analysis. *Psychological Medicine*. Published online July 17, 2023:1-24. doi:10.1017/S0033291723001678

4. Vargas T, Lam PH, Azis M, Osborne KJ, Lieberman A, Mittal VA. Childhood Trauma and Neurocognition in Adults With Psychotic Disorders: A Systematic Review and Meta-analysis. *Schizophr Bull*. 2019;45(6):1195-1208. doi:10.1093/schbul/sby150

5. Miskowiak KW, Hansen KB, Mariegaard J, Kessing LV. Association between childhood trauma, cognition, and psychosocial function in a large sample of partially or fully remitted patients with bipolar disorder and healthy participants. *Int J Bipolar Disord*. 2023;11(1):31. doi:10.1186/s40345-023-00311-w

6. Little B, Anwyll M, Norsworthy L, Corbett L, Schultz-Froggatt M, Gallagher P. Processing speed and sustained attention in bipolar disorder and major depressive disorder: A systematic review and meta-analysis. *Bipolar Disorders*. 2024;26(2):109-128. doi:10.1111/bdi.13396

7. Cotrena C, Damiani Branco L, Ponsoni A, Samamé C, Milman Shansis F, Paz Fonseca R. Executive functions and memory in bipolar disorders I and II: new insights from meta‐analytic results. *Acta Psychiatr Scand*. 2020;141(2):110-130. doi:10.1111/acps.13121

8. Rosa M, Scassellati C, Cattaneo A. Association of childhood trauma with cognitive domains in adult patients with mental disorders and in non-clinical populations: a systematic review. *Front Psychol*. 2023;14:1156415. doi:10.3389/fpsyg.2023.1156415

9. Samamé C, Durante P, Cattaneo B, Aprahamian I, Strejilevich S. Efficacy of cognitive remediation in bipolar disorder: systematic review and meta-analysis of randomized controlled trials. *Psychological Medicine*. 2023;53(12):5361-5373. doi:10.1017/S0033291723001897

10. Saldarini F, Gottlieb N, Stokes PRA. Neural correlates of working memory function in euthymic people with bipolar disorder compared to healthy controls: A systematic review and meta-analysis. *Journal of Affective Disorders*. 2022;297:610-622. doi:10.1016/j.jad.2021.10.084

11. Diamond A. Executive Functions. *Annu Rev Psychol*. 2013;64:135-168. doi:10.1146/annurev-psych-113011-143750

12. Raucher-Chéné D, Achim AM, Kaladjian A, Besche-Richard C. Verbal fluency in bipolar disorders: A systematic review and meta-analysis. *J Affect Disord*. 2017;207:359-366. doi:10.1016/j.jad.2016.09.039

13. Fares-Otero NE, Schalinski I. Social cognition in maltreated individuals: Do type and timing of maltreatment matter? *European Neuropsychopharmacology*. 2024;81:38-40. doi:10.1016/j.euroneuro.2023.12.011

14. Kilford EJ, Garrett E, Blakemore SJ. The development of social cognition in adolescence: An integrated perspective. *Neuroscience & Biobehavioral Reviews*. 2016;70:106-120. doi:10.1016/j.neubiorev.2016.08.016

15. Jiménez E, Solé B, Arias B, et al. Impact of childhood trauma on cognitive profile in bipolar disorder. *Bipolar Disorders*. 2017;19(5):363-374. doi:10.1111/bdi.12514

16. Ehrlich TJ, Kim H, Ryan KA, et al. Childhood trauma relates to worse memory functioning in bipolar disorder. *Journal of Affective Disorders*. 2023;333:377-383. doi:10.1016/j.jad.2023.04.056

17. Meyers JE, Meyers KR. *Rey Complex Figure Test and Recognition Trial (RCFT)*. Psychological Assessment Resources Odessa, FL; 1995.

18. Bücker J, Kozicky J, Torres IJ, et al. The impact of childhood trauma on cognitive functioning in patients recently recovered from a first manic episode: Data from the Systematic Treatment Optimization Program for Early Mania (STOP-EM). *Journal of Affective Disorders*. 2013;148(2-3):424-430. doi:10.1016/j.jad.2012.11.022

19. Tiffin J, Asher EJ. The Purdue Pegboard: norms and studies of reliability and validity. *Journal of Applied Psychology*. 1948;32(3):234-247. doi:10.1037/h0061266

20. Rios U, Moya PR, Urrejola O, et al. History of child abuse among patients with bipolar disorders. *REVISTA MEDICA DE CHILE*. 2020;148(2):204-210.

21. Muñoz-Neira C, Henríquez Ch F, Ihnen J J, Sánchez C M, Flores M P, Slachevsky Ch A. Psychometric properties and diagnostic usefulness of the Addenbrooke’s Cognitive Examination-revised in a Chilean elderly sample. *Revista médica de Chile*. 2012;140(8):1006-1013. doi:10.4067/S0034-98872012000800006

22. Strauss E, Sherman EMS, Spreen O. *A Compendium of Neuropsychological Tests: Administration, Norms, and Commentary, 3rd Ed.* Oxford University Press; 2006:xvii, 1216.

23. Oymak Yenilmez D, Atagun MI, Keles Altun I, et al. Relationship between Childhood Adversities, Emotion Dysregulation and Cognitive Processes in Bipolar Disorder and Recurrent Depressive Disorder. *Turkish Journal of Psychiatry*. Published online 2019. doi:10.5080/u23415

24. Mowlds W, Shannon C, McCusker CG, et al. Autobiographical memory specificity, depression, and trauma in bipolar disorder. *Br J Clin Psychol*. 2010;49(Pt 2):217-233. doi:10.1348/014466509X454868

25. Larsen EM, Ospina LH, Cuesta-Diaz A, et al. Effects of childhood trauma on adult moral decision-making: Clinical correlates and insights from bipolar disorder. *J Affective Disord*. 2019;244:180-186. doi:10.1016/j.jad.2018.10.002

26. Morán-Kneer J, Ríos U, Costa-Cordella S, et al. Childhood trauma and social cognition in participants with bipolar disorder: The moderating role of attachment. *Journal of Affective Disorders Reports*. 2022;9:100359. doi:10.1016/j.jadr.2022.100359

27. Vreeker A, Abramovic L, Boks MPM, et al. The relationship between brain volumes and intelligence in bipolar disorder. *J Affective Disord*. 2017;223:59-64. doi:10.1016/j.jad.2017.07.009

28. Quidé Y, Cohen-Woods S, O’Reilly N, Carr VJ, Elzinga BM, Green MJ. Schizotypal personality traits and social cognition are associated with childhood trauma exposure. *The British Journal of Clinical Psychology*. 2018;57(4):397-419. doi:10.1111/bjc.12187

29. Ríos U, Morán J, Hermosilla J, et al. The interaction of the oxytocin receptor gene and child abuse subtypes on social cognition in euthymic patients with bipolar disorder type I. *Front Psychiatry*. 2023;14:1151397. doi:10.3389/fpsyt.2023.1151397

30. Vaughn-Coaxum RA, Merranko J, Birmaher B, et al. Longitudinal course of depressive symptom severity among youths with bipolar disorders: Moderating influences of sustained attention and history of child maltreatment. *Journal of Affective Disorders*. 2021;282:261-271. doi:10.1016/j.jad.2020.12.078

31. Aas M, Steen NE, Agartz I, et al. Is cognitive impairment following early life stress in severe mental disorders based on specific or general cognitive functioning? *Psychiatry Research*. 2012;198(3):495-500. doi:10.1016/j.psychres.2011.12.045

32. Keefe RSE, Fox KH, Davis VG, et al. The Brief Assessment of Cognition In Affective Disorders (BAC-A):performance of patients with bipolar depression and healthy controls. *J Affect Disord*. 2014;166:86-92. doi:10.1016/j.jad.2014.05.002

33. Kaufman AS. Kaufman brief intelligence test: KBIT. *Circle Pines, MN: American Guidance Service*. Published online 1990.

34. Nelson HE, O’Connell A. Dementia: The Estimation of Premorbid Intelligence Levels Using the New Adult Reading Test. *Cortex*. 1978;14(2):234-244. doi:10.1016/S0010-9452(78)80049-5

35. Berger H, Kunst M, Deelman BG. [Dementia and the Dutch Reading Tests for Adults]. *Tijdschr Gerontol Geriatr*. 1996;27(6):250-254.

36. Randolph C, Tierney MC, Mohr E, Chase TN. The Repeatable Battery for the Assessment of Neuropsychological Status (RBANS): preliminary clinical validity. *J Clin Exp Neuropsychol*. 1998;20(3):310-319. doi:10.1076/jcen.20.3.310.823

37. Wechsler D. Wechsler Abbreviated Scale of Intelligence--Second Edition. Published online June 11, 2018. doi:10.1037/t15171-000

38. Ryan JJ, Rosenberg SJ. Validity of the Verbal IQ as a short form of the Wechsler Adult Intelligence Scale-Revised. *J Clin Psychol*. 1984;40(1):306-308. doi:10.1002/1097-4679(198401)40:1<306::aid-jclp2270400157>3.0.co;2-s

39. Wechsler D 1896-1981., Psychological Corporation. *WAIS-III : Administration and Scoring Manual : Wechsler Adult Intelligence Scale*. 3rd ed. Psychological Corporation; 1997.

40. Cambridge Cognition. The Cambridge Neuropsychological Testing Automated Battery (CANTAB). Published online 2006.

41. Rapid Visual Information Processing (RVP). Cambridge Cognition. Accessed July 21, 2024. https://cambridgecognition.com/rapid-visual-information-processing-rvp/

42. Conners CK. *Conners’ Continuous Performance Test (CPT-II). Technical Guide and Software Manual.* Multi-Health Systems; 2002.

43. Pino O, Guilera G, Gómez J, Rojo JE, Vellejo J, Purdon SE. [A brief scale to assess cognitive impairment in psychiatric patients]. *Psicothema*. 2006;18(3):447-452.

44. Adjutant General’s Office, Classification and Replacement Branch, Personnel Research Section. Army Individual Test Battery. Published online July 7, 2014. doi:10.1037/t31500-000

45. Mowlds W, Shannon C, McCusker CG, et al. Autobiographical memory specificity, depression, and trauma in bipolar disorder. *Br J Clin Psychol*. 2010;49(Pt 2):217-233. doi:10.1348/014466509X454868

46. Ros L, Romero D, Ricarte JJ, Serrano JP, Nieto M, Latorre JM. Measurement of overgeneral autobiographical memory: Psychometric properties of the autobiographical memory test in young and older populations. *PLoS ONE*. 2018;13(4):e0196073. doi:10.1371/journal.pone.0196073

47. Delis DC, Kramer JH, Kaplan E, Ober BA. *California Verbal Learning Test--Second Edition*. Assessment; 2016. Accessed July 6, 2024. https://doi.apa.org/doi/10.1037/t15072-000

48. Paired Associates Learning (PAL). Cambridge Cognition. Accessed July 21, 2024. https://cambridgecognition.com/paired-associates-learning-pal/

49. Pattern Recognition Memory (PRM). Cambridge Cognition. Accessed July 21, 2024. https://cambridgecognition.com/pattern-recognition-memory-prm/

50. Rey André. *L’examen clinique en psychologie*. [1. éd.]. Presses universitaires de France; 1958.

51. Anil AE, Kivircik BB, Batur S, et al. The Turkish Version of the Auditory Consonant Trigram Test as a Measure of Working Memory: A Normative Study. *The Clinical Neuropsychologist*. 2003;17(2):159-169. doi:10.1076/clin.17.2.159.16510

52. Spatial Working Memory (SWM). Cambridge Cognition. Accessed July 21, 2024. https://cambridgecognition.com/spatial-working-memory-swm/

53. Patton JH, Stanford MS, Barratt ES. Factor structure of the barratt impulsiveness scale. *Journal of Clinical Psychology*. 1995;51(6):768-774. doi:10.1002/1097-4679(199511)51:6<768::AID-JCLP2270510607>3.0.CO;2-1

54. Lima IMM, Malloy-Diniz LF, de Miranda DM, Da Silva AG, Neves FS, Johnson SL. Integrative Understanding of Familial Impulsivity, Early Adversity and Suicide Risk. *Front Psychol*. 2017;8. doi:10.3389/fpsyg.2017.02240

55. Intra-Extra Dimensional Set Shift (IED). Cambridge Cognition. Accessed July 21, 2024. https://cambridgecognition.com/intra-extra-dimensional-set-shift-ied/

56. One Touch Stockings of Cambridge (OTS). Cambridge Cognition. Accessed July 21, 2024. https://cambridgecognition.com/one-touch-stockings-of-cambridge-ots/

57. Benton AL, Sivan AB. Problems and conceptual issues in neuropsychological research in aging and dementia. *J Clin Neuropsychol*. 1984;6(1):57-63. doi:10.1080/01688638408401196

58. Votruba KL, Langenecker SA. Factor structure, construct validity, and age- and education-based normative data for the Parametric Go/No-Go Test. *Journal of Clinical and Experimental Neuropsychology*. 2013;35(2):132-146. doi:10.1080/13803395.2012.758239

59. Stroop JR. Studies of interference in serial verbal reactions. *Journal of Experimental Psychology*. 1935;18:643-662. doi:10.1037/h0054651

60. Periáñez JA, Lubrini G, García-Gutiérrez A, Ríos-Lago M. Construct Validity of the Stroop Color-Word Test: Influence of Speed of Visual Search, Verbal Fluency, Working Memory, Cognitive Flexibility, and Conflict Monitoring. *Archives of Clinical Neuropsychology*. 2021;36(1):99-111. doi:10.1093/arclin/acaa034

61. Borkowski JG, Benton AL, Spreen O. Word fluency and brain damage. *Neuropsychologia*. 1967;5(2):135-140. doi:10.1016/0028-3932(67)90015-2

62. Heaton RK. *Wisconsin Card Sorting Test*. Psychological Assessment Resources; 1981.

63. Nyhus E, Barceló F. The Wisconsin Card Sorting Test and the cognitive assessment of prefrontal executive functions: a critical update. *Brain Cogn*. 2009;71(3):437-451. doi:10.1016/j.bandc.2009.03.005

64. Mayer JD, Salovey P, Caruso DR, Sitarenios G. Measuring emotional intelligence with the MSCEIT V2.0. *Emotion*. 2003;3(1):97-105. doi:10.1037/1528-3542.3.1.97

65. McDonald S, Flanagan S, Rollins J, Kinch J. TASIT: A New Clinical Tool for Assessing Social Perception After Traumatic Brain Injury. *The Journal of Head Trauma Rehabilitation*. 2003;18(3):219.

66. Bora E, Berk M. Theory of mind in major depressive disorder: A meta-analysis. *J Affect Disord*. 2016;191:49-55. doi:10.1016/j.jad.2015.11.023

67. Değirmencioğlu B, Alptekin K, Akdede BB, et al. [The Validity and Reliability Study of the Dokuz Eylül Theory of Mind Index (DEZİKÖ) in Patients with Schizophrenia]. *Turk Psikiyatri Derg*. 2018;29(3):193-201.

68. Takım U, Sarı S, Gokcay H. “The Relationship Between Childhood Traumas and Social Cognition Through Theory of Mind and Alexithymia in Bipolar Disorder.” *Psychol Rep*. Published online August 4, 2024:332941241269549. doi:10.1177/00332941241269549

69. Klein HS, Springfield CR, Bass E, et al. Measuring mentalizing: A comparison of scoring methods for the hinting task. *International Journal of Methods in Psychiatric Research*. 2020;29(2):e1827. doi:10.1002/mpr.1827

70. Bru-Luna LM, Martí-Vilar M, Merino-Soto C, Cervera-Santiago JL. Emotional Intelligence Measures: A Systematic Review. *Healthcare (Basel)*. 2021;9(12):1696. doi:10.3390/healthcare9121696

71. Emotion Recognition Task (ERT). Cambridge Cognition. Accessed July 21, 2024. https://cambridgecognition.com/emotion-recognition-task-ert/

72. Green P, Allen L. *The Emotional Perception Test*. Durham, NC: CogniSyst Inc; 1997.

73. Young AW, Perrett DI, Calder AJ, Sprengelmeyer R, Ekman P. *Facial Expressions of Emotion: Stimuli and Tests (FEEST)*.; 2002. https://api.semanticscholar.org/CorpusID:142031276

74. Levin HS, Hamsher K de S, Benton AL. A Short Form of the Test of Facial Recognition for Clinical Use. *The Journal of Psychology*. 1975;91(2):223-228. doi:10.1080/00223980.1975.9923946

75. Wells G, Wells G, Shea B, et al. The Newcastle-Ottawa Scale (NOS) for Assessing the Quality of Nonrandomised Studies in Meta-Analyses. In: ; 2014. Accessed April 30, 2023. https://www.semanticscholar.org/paper/The-Newcastle-Ottawa-Scale-(NOS)-for-Assessing-the-Wells-Wells/c293fb316b6176154c3fdbb8340a107d9c8c82bf

76. Epstein S, Roberts E, Sedgwick R, et al. Poor school attendance and exclusion: a systematic review protocol on educational risk factors for self-harm and suicidal behaviours. *BMJ Open*. 2018;8(12):e023953. doi:10.1136/bmjopen-2018-023953

77. Herzog R, Álvarez-Pasquin MJ, Díaz C, Del Barrio JL, Estrada JM, Gil Á. Are healthcare workers’ intentions to vaccinate related to their knowledge, beliefs and attitudes? a systematic review. *BMC Public Health*. 2013;13(1):154. doi:10.1186/1471-2458-13-154

78. Wickersham A, Leightley D, Archer M, Fear NT. The association between paternal psychopathology and adolescent depression and anxiety: A systematic review. *J Adolesc*. 2020;79:232-246. doi:10.1016/j.adolescence.2020.01.007

79. Mertz D, Kim TH, Johnstone J, et al. Populations at risk for severe or complicated influenza illness: systematic review and meta-analysis. *BMJ*. 2013;347:f5061. doi:10.1136/bmj.f5061
